# The landscape of responses to neoadjuvant immunotherapy in resectable Kirsten rat sarcoma viral oncogene homolog‐mutant lung adenocarcinoma: Clinical heterogeneity and correlative immunologic analysis

**DOI:** 10.1002/ctm2.70670

**Published:** 2026-04-20

**Authors:** Sikai Wu, Jiheng Niu, Xiaowei Chen, Jinfei Li, Quanying Tang, Zhenlin Yang, Shugeng Gao

**Affiliations:** ^1^ Department of Thoracic Surgery, National Cancer Center/National Clinical Research Center for Cancer/Cancer Hospital Chinese Academy of Medical Sciences and Peking Union Medical College Beijing China; ^2^ School of Basic Medical Sciences Tsinghua University Beijing China; ^3^ Department of Thoracic Surgery Shandong Provincial Hospital Affiliated to Shandong First Medical University Jinan Shandong China

**Keywords:** KRAS‐mutant lung adenocarcinoma, neoadjuvant immunotherapy, single‐cell RNA sequencing, tumour microenvironment

## Abstract

**Background:**

Kirsten rat sarcoma viral oncogene homolog (KRAS)‐mutant lung adenocarcinoma (LUAD) typically demonstrates limited response to neoadjuvant immunotherapy (NIT). Elucidating the immune determinants that differentiate responders from non‐responders was critical for optimizing immunotherapy strategies. This study aimed to characterize the tumour microenvironment features of KRAS‐mutant LUAD following neoadjuvant programmed death protein 1 (PD‐1) inhibitor therapy by integrating clinical outcomes with single‐cell RNA sequencing (scRNA‐seq).

**Methods:**

A total of 143 patients with resectable LUAD were consecutively enrolled in this study, including 106 cases in the KRAS‐wildtype cohort and 37 cases in the KRAS‐mutant cohort. We systematically compared the pathological response rates, survival outcomes and recurrence patterns between the two cohorts. We performed scRNA‐seq on tumour specimens from 234 real‐world patients with non‐small cell lung cancer. From this cohort, 48 LUAD cases were identified and stratified by KRAS mutation status (13 KRAS‐mutant and 35 KRAS‐wildtype patients). Cellular compositions, transcriptional features and intercellular communication networks were analysed.

**Results:**

Clinical analysis revealed that the KRAS‐mutant group exhibited significantly poorer pathological responses (*p* = .032) and inferior long‐term survival compared to the KRAS‐wildtype group. We identified an immunosuppressive tumour necrosis factor receptor superfamily member 4 (TNFRSF4)‐expressing regulatory T‐cell (CD4T_Treg_TNFRSF4) subset enriched in non‐responders, whereas responders showed increased frequencies of T helper 1 cells (Th1 cells) and a previously unrecognized exhausted‐like B‐cell state (Bex). Bex cells displayed impaired metabolic activity yet retained antigen presentation potential and showed extensive cellular interactions with Th1 cells, suggesting a supportive role in Th1‐mediated antitumour immunity.

**Conclusion:**

KRAS‐mutant patients exhibited significantly poorer pathological responses, and KRAS‐mutant status may independently predict survival outcomes after NIT in LUAD patients. Additionally, our study unveiled the cellular and molecular architecture underlying differential responses to NIT in KRAS‐mutant LUAD, emphasizing the opposing roles of immunosuppressive Tregs and synergistic Bex–Th1 networks.

**Highlights:**

1. KRAS‐mutant LUAD patients exhibit inferior pathological responses and survival after neoadjuvant immunotherapy compared to KRAS‐wildtype patients.
2. A CD4T_Treg_TNFRSF4 subset is enriched in non‐responders, defining an immunosuppressive microenvironment.
3. Responders are characterized by a synergistic network between Th1 cells and a novel exhausted‐like B‐cell (Bex) state.
4. The balance between immunosuppressive Tregs and the Th1/Bex axis determines therapeutic efficacy in KRAS‐mutant LUAD.

## Graphical abstract

- KRAS‐mutant LUAD patients exhibit inferior pathological responses and survival after neoadjuvant immunotherapy compared to KRAS‐wildtype patients.
- A CD4T_Treg_TNFRSF4 subset is enriched in non‐responders, defining an immunosuppressive microenvironment.
- Responders are characterized by a synergistic network between Th1 cells and a novel exhausted‐like B‐cell (Bex) state.
- The balance between immunosuppressive Tregs and the Th1/Bex axis determines therapeutic efficacy in KRAS‐mutant LUAD.

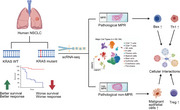

## 1 INTRODUCTION

Non‐small cell lung cancer (NSCLC), with lung adenocarcinoma (LUAD) as the predominant subtype, accounts for over 85% of all lung cancer cases. This malignancy exhibits remarkable molecular heterogeneity, which exerts a profound impact on the selection and efficacy of therapeutic regimens.^1^ Immune checkpoint inhibitors (ICIs) directed against programmed cell death protein 1 (PD‐1) and programmed cell death 1‐ligand 1 (PD‐L1) have reshaped the treatment landscape for NSCLC. In particular, neoadjuvant or perioperative chemoimmunotherapy has emerged as a standard approach for resectable locally advanced NSCLC.^2^, ^3^ Neoadjuvant immunotherapy (NIT) functions by leveraging elevated tumour antigen levels to enhance immune priming, thereby improving immune surveillance of micrometastatic disease.^4^ Therapeutic efficacy can be assessed at surgical resection by quantifying the degree of pathological tumour regression using standardized thresholds, namely, pathologic complete response (pCR) and major pathological response (MPR).^5^, ^6^ In the randomized phase III CheckMate 816 trial,^2^ patients with resectable NSCLC who received neoadjuvant nivolumab combined with chemotherapy demonstrated a significant improvement in overall survival (OS) compared to those receiving chemotherapy alone. The trial established neoadjuvant chemoimmunotherapy as a superior approach for resectable NSCLC.

Kirsten rat sarcoma viral oncogene homolog (KRAS) mutations occur in approximately 20%–30% of LUAD cases, representing the most common oncogenic driver.^7^ Before the advent of KRAS G12C‐specific inhibitors, which yield objective response rates (ORRs) of 37%–43%,^8^ KRAS‐mutant patients were mainly treated with chemoimmunotherapy or immunotherapy alone. Recent investigations have demonstrated that KRAS mutations correlate with distinct immune‐related characteristics, such as tumour mutational burden (TMB), PD‐L1 expression levels and the degree of immune cell infiltration within the tumour microenvironment (TME).^9^, ^10^, ^11^ Furthermore, previous studies have indicated that within KRAS‐mutant NSCLC, concurrent serine/threonine kinase 11 (STK11) mutations may negatively impact clinical outcomes following neoadjuvant ICIs, potentially by promoting T‐cell chronic stimulation and tissue residency.^12^ This clinical and molecular diversity underscores that KRAS mutation is not a uniform biomarker but rather a spectrum of immune‐modulatory contexts. Critically, prior research has predominantly focused on advanced‐stage cohorts, leaving a significant gap in the understanding of the unique immunobiological features of resectable KRAS‐mutant LUAD under the selective pressure of NIT.^7^ It remains unclear why some patients achieve an MPR while others experience rapid disease progression, and whether KRAS status itself dictates a distinct trajectory of immune evasion in locally advanced disease. Defining the immune determinants that distinguish responders from non‐responders is therefore essential for optimizing immunotherapeutic strategies.

To address these gaps, we characterized the TME of KRAS‐mutant LUAD after neoadjuvant PD‐1 blockade by integrating clinical data, single‐cell RNA sequencing (scRNA‐seq) and multiplex immunohistochemistry (mIHC). We identified a novel ‘exhausted‐like’ B‐cell (Bex) population that, unexpectedly, functions as a positive predictor of response via crosstalk with T helper 1 cells (Th1 cells), challenging the conventional view of immune exhaustion. We pinpointed a specific tumour necrosis factor receptor superfamily member 4 (TNFRSF4)‐expressing Treg subset (CD4T_Treg_TNFRSF4) as a critical determinant of non‐response, providing a potential mechanistic explanation for the inferior pathological outcomes observed in KRAS‐mutant patients. By elucidating these specific ‘responders versus non‐responders’ immune determinants, our study moves beyond general KRAS biology to offer actionable insights for optimizing neoadjuvant strategies.

## 2 METHODS AND MATERIALS

### 2.1 Study design and patient selection

We retrospectively reviewed data from 550 patients who received NIT followed by radical resection at the Chinese Academy of Medical Sciences Cancer Hospital (CHCAMS) between July 2017 and October 2025. Eligibility required (1) pathologically confirmed stage II‐III LUAD; (2) completion of NIT followed by curative‐intent surgery; and (3) availability of complete baseline records. Patients were excluded for the following reasons: non‐LUAD histology (*n* = 342), palliative resection (*n* = 3), incomplete clinical data (*n* = 41) or tumours harbouring epidermal growth factor receptor (EGFR) mutations or other non‐KRAS driver alterations (*n* = 21). To ensure accurate staging and resectability assessment, all cases underwent systematic multidisciplinary evaluation before and after treatment. Pre‐NIT evaluation included contrast‐enhanced chest computed tomography (CT), whole‐body positron emission tomography‐CT, brain magnetic resonance imaging (MRI) and pathological confirmation via endobronchial ultrasound/transbronchial needle aspiration or CT‐guided lung biopsy. A multidisciplinary team comprising thoracic surgeons, medical oncologists, radiologists and pathologists reviewed all imaging and pathology findings to confirm clinical stage.

From a prospective cohort of 234 patients who underwent resection after neoadjuvant therapy, tumour specimens were subjected to scRNA‐seq. Within this cohort, we selected 48 patients with LUAD for in‐depth characterization of the tumour‐immune microenvironment using single‐cell transcriptomics. These LUAD cases were categorized into two groups: KRAS‐mutant (*n* = 13) and wildtype (*n* = 35) (Figure 2A).

### 2.2 Pre‐operative neoadjuvant therapy and pathological assessment

All patients completed two to four cycles (21 days per cycle) of NIT, which included a PD‐1 inhibitor (such as camrelizumab, nivolumab, sintilimab, tislelizumab or pembrolizumab) combined with platinum‐based chemotherapy (cisplatin 75 mg/m^2^ or carboplatin at AUC 5) plus nab‐paclitaxel 260 mg/m^2^. Patients were stratified by KRAS mutation status into KRAS‐mutant and KRAS‐wildtype groups. Tumour staging followed the eighth edition of the IASLC TNM classification.^13^ Radiological responses were evaluated according to RECIST v1.1^5^ and classified as complete response, partial response (PR), stable disease or progressive disease (PD). Pathological assessment was performed on biopsy and surgical specimens using standardized protocols. pCR was defined as no residual viable tumour cells in the primary tumour bed and lymph nodes and MPR as ≤10% viable tumour cells. All pathological responses were independently reviewed by two experienced pathologists.

### 2.3 Perioperative outcomes, survival and recurrence

Demographic and clinical variables encompassed age, sex, surgical approach, smoking history, clinical/pathological stage, family history of cancer, tumour location, body mass index and type of resection. Pre‐operative comorbidities were quantified using the age‐adjusted Charlson Comorbidity Index (aCCI), and postoperative complications were graded by the Clavien–Dindo system.^14^ OS was calculated from surgery to death or last follow‐up. Recurrence‐free survival (RFS) spanned from surgery to confirmed recurrence or last follow‐up. Locoregional recurrence (LR) was defined as relapse within mediastinal lymph nodes, the bronchial stump or the ipsilateral lung. Distant recurrence (DR) referred to recurrence in supraclavicular lymph nodes, pleura, contralateral lung or extracranial/extrathoracic organs (e.g., brain, liver or other distant sites).

### 2.4 Biospecimen collection and processing

Tumour tissues were freshly obtained from patients undergoing lung resection at the hospital. Tumour tissues for scRNA‐seq were collected as fresh samples through intraoperative prospective acquisition at the time of radical resection. Surgical tumour specimens and matched adjacent normal tissues (approximately 2 cm^3^ each) were collected from resection materials and processed within 30 min after surgery to remove necrotic regions and blood clots (Figure 2A). To ensure sample quality, the following measures were implemented: (1) Tumour beds were identified by experienced pathologists through gross inspection and palpation according to IASLC recommendations for pathological response assessment. To minimize inclusion of non‐tumour‐related inflammatory cells, scRNA‐seq samples were taken from the inner zone of the tumour bed. (2) Based on the most recent contrast‐enhanced CT imaging, a multidisciplinary team evaluation was performed to avoid radiologically evident necrotic areas and to precisely target sampling within the solid, viable tumour components. Pre‐treatment and pre‐operative CT scans were referenced to assist in gross identification and demarcation of the tumour bed location and extent during macroscopic examination. Furthermore, for a subset of cases, intraoperative frozen section analysis was employed for immediate pathological verification, thereby confirming the viability of the sampled tissue at the earliest point ex vivo. (3) For histopathological correlation, formalin‐fixed paraffin‐embedded (FFPE) blocks were generated from tissue regions immediately adjacent to the scRNA‐seq sampling sites within the same resection specimen. Haematoxylin and eosin staining of these FFPE sections subsequently verified the presence of treatment‐related regression features and residual viable tumour cells. All fresh tissue samples were placed in preservation buffer (Miltenyi, 130‐100‐008) immediately after collection, maintained on ice and transported to Peking University for processing. These samples were designated for scRNA‐seq and FFPE analyses. It is noted that neither core needle biopsies nor endobronchial biopsies were included in the scRNA‐seq cohort.

### 2.5 Tissue dissociation and scRNA‐seq

These methods have been published in detail previously.^15^ Fresh surgical tumour specimens were minced into approximately 1 mm^3^ fragments in RPMI‐1640 medium (Gibco, 11875093) containing 10% foetal bovine serum (FBS; Gibco, 10099141). Tissue fragments were enzymatically digested for 60 min at 37°C on a rotator using a Tumour Dissociation Kit (Miltenyi, 130‐095‐929). The digested suspension was subsequently filtered through a 100 µm MACS SmartStrainer (Miltenyi, 130‐110‐917) and centrifuged at 300 × *g* for 8 min. Following centrifugation, the supernatant was removed and the cell pellet was resuspended in red blood cell lysis buffer (TIANDZ, 90309‐100) for 5 min on ice to eliminate erythrocytes. After washing with PBS (Gibco, 10010023), cells were resuspended in sorting buffer (PBS with 2% FBS). Viability was assessed by staining with 7‐AAD. Cell suspensions were then adjusted to a concentration of 700–1200 cells/µL, and only those with >80% viability were used for subsequent single‐cell sequencing. A total of 18 000 cells per sample were targeted for library construction using the 10× Genomics Chromium Single Cell 5′ Kit in conjunction with the Human T‐Cell Receptor Library Construction Kit, strictly following the manufacturer's protocols for all subsequent steps. The purified libraries were sequenced on an Illumina NovaSeq 6000 platform for paired‐end 150‐base pair reads.

### 2.6 scRNA‐seq data processing and quality control

scRNA‐seq reads were processed using the CellRanger pipeline (v6.1.2) and aligned to the GRCh38 human reference genome. Downstream analyses were conducted with Scanpy (v1.9.3).^16^ Quality control filters were applied to retain high‐quality cells: (1) total UMI counts between 1200 and 40 000; (2) 200–6000 genes detected per cell; and (3) mitochondrial gene content below 5%. Genes expressed in fewer than three cells were removed from further analysis.

### 2.7 Cell clustering and annotation

Single‐cell clustering and annotation were performed using Scanpy (v1.9.3).^16^ Following total‐count normalization and regression of total counts per cell and mitochondrial gene percentage, 2000 highly variable genes were identified for downstream analyses. Principal component analysis was conducted, and the top 50 components were used for dimensionality reduction and clustering. To mitigate batch effects and enhance the consistency of visualization and clustering, we performed batch effect correction using the scanpy.external.harmonypy() function. Uniform manifold approximation and projection (UMAP) was applied for low‐dimensional embedding, and graph‐based clustering using the Leiden algorithm was performed, with initial clustering executed under default settings. Major cell types were assigned based on canonical markers, including *CD2*, *CD3D*, *CD3E* (T/NK cells), *CD19*, *CD79A*, *MS4A1*, *JCHAIN* (B/plasmsa cells), *CD68*, *LYZ* (myeloid cells), *PECAM1*, *VWF*, *ENG* (endothelial cells), *EPCAM*, *KRT17*, *KRT19* (epithelial cells) and *COL1A1*, *ACTA2*, *DCN* (fibroblasts). To further resolve subpopulations, epithelial cells, CD4^+^ T cells and B cells underwent a second round of clustering after exclusion of cells expressing residual off‐target markers.

### 2.8 CNV estimation and identification of malignant cells

To distinguish malignant epithelial cells, copy number variations (CNVs) were inferred using the infercnvpy algorithm (v0.2.0; https://github.com/icbi‐lab/infercnvpy). Fibroblasts served as a normal reference, and the analysis was performed using default settings. Concurrently, we examined the expression of known marker genes for normal epithelial cells (e.g., ciliated cells, alveolar cells) across the clusters to ensure the specificity of the malignant cell annotation. Notably, classic ciliated cell marker genes (e.g., *FOXJ1*, *TPPP3*) were not expressed within the malignant epithelial cell cluster, confirming that this population was not contaminated by normal respiratory epithelial cells. All subsequent analyses were performed on the CNV‐defined malignant epithelial cells. CNV scores for individual cells were computed with the infercnvpy.tl.cnv_score function. Malignant epithelial cells exhibit a significantly higher number of CNV scores than normal epithelial cells.

### 2.9 Enrichment analysis and GSEA analysis

Gene set enrichment analysis (GSEA) in Python (version 1.1.1)^17^ was employed for enrichment analyses. Differentially expressed genes (DEGs) (fold change ≥ 2, *p* < .05) underwent gene ontology (GO) and Kyoto Encyclopedia of Genes and Genomes (KEGG) pathway enrichment analyses, with an adjusted *p*‐value <.05 considered significant. GSEA was performed on the single‐cell transcriptomic data using default settings. Enrichment was deemed significant if the following criteria were met: normalized enrichment score >1, nominal *p* < .05 and false discovery rate *q* < .25.

### 2.10 Single‐cell regulatory network inference and clustering (SCENIC)

The pySCENIC Python package (version 0.12.1)^18^ was utilized for single‐cell regulatory network inference and clustering analysis. The co‐expression modules linking transcription factors (TFs) to their candidate target genes were initially constructed using GRNBoost2. The RcisTarget algorithm was then applied to identify enriched motifs within each module. Finally, AUCell was used to quantify regulon activity in individual cells, generating an activation score for each TF per cell. All analyses were performed using default parameters.

### 2.11 Cell–cell interaction analysis

Cell–cell interactions among subsets were inferred using the LIANA^+^ framework,^19^ employing the CellPhoneDB ligand–receptor method. Ligand–receptor pairs with statistical significance (*p* < .01) were retained, and these significant interactions were selected for downstream visualization.

### 2.12 Overall survival and recurrence‐free survival analysis

Receiver operating characteristic curves were generated in Python using the sklearn package (v1.1.3), with cancer recurrence or death as the categorical outcome. The optimal cut‐off value was identified by maximizing Youden's *J* statistic. Hazard ratios (HRs) and corresponding *p*‐values were derived from Cox proportional hazards models. Kaplan–Meier survival curves were plotted with the lifelines package (v0.28.0).

### 2.13 Single‑cell metabolic activity analysis

Metabolic activities in B‐cell subsets were evaluated with the scMetabolism R package (v0.2.1)^20^ according to its default workflow and parameters.

### 2.14 Gene signature scoring for single‐cell analysis

Gene sets were obtained from the GO database (2025) and the KEGG_2021_Human database. Signature scores for single cells were computed using the scanpy.tl.score_genes function.

### 2.15 HdWGCNA analysis

To identify Th1 cell‐associated key genes in metastatic samples, high‐dimensional weighted gene co‐expression network analysis (hdWGCNA)^21^ was employed. A gene expression correlation matrix was constructed, followed by weighted co‐expression network building and module detection using default parameters. Module‐trait analysis was then applied to identify modules significantly correlated with Th1 cells. Hub genes were identified from the significant modules based on intramodular connectivity. Module 3 contained 832 hub genes, which were defined as key Th1‐associated genes.

### 2.16 Pseudo‐time analyses and trajectory inference

Pseudo‐time analyses of B‐cell subsets were performed using the R Monocle2 package (version 2.24.0)^22^ with default parameters. For trajectory analysis, genes detected in >1% of cells were retained. Cells were ordered along pseudo‐time using DEGs defined by a fold change ≥2 and *p* < .05. Dimensionality reduction was carried out with the DDRTree method, and differentiation trajectories were subsequently inferred under default settings.

### 2.17 Ligand–receptor interaction analysis

Intercellular interactions among the CD4T_Treg_TNFRSF4, CD4T_Th1_NFKB1 and Bex_NFKB1 subsets were interrogated using the CellChat R package (v1.6.1)^23^ with default parameters. Biologically relevant cell–cell communications were identified based on a curated ligand–receptor interaction database. Statistically significant interactions were defined as those with a *p‐*value <.05.

### 2.18 External dataset validation

This study utilized the following publicly available single‐cell and bulk transcriptome datasets for independent validation. The publicly available datasets GSE50081^24^ and GSE280232^12^ used in the analysis are accessible through the NCBI GEO database (https://www.ncbi.nlm.nih.gov/geo/). The gene signatures were constructed by selecting the top 30 most significantly up‐regulated DEGs calculated during the Scanpy pipeline. For the bulk RNA‐seq database (GSE50081),^24^ single‐sample GSEA using the GSVA R package (version 1.46.0) was performed to quantify the enrichment levels of the gene signatures in individual samples. Samples diagnosed as LUAD were selected for downstream survival analysis. For the scRNA‐seq database GSE280232,^12^ we re‐analysed the expression data using the Scanpy pipeline following the same protocol. Based on the single‐cell data, T cells, CD4^+^ T cells and Tregs were annotated in a stepwise manner. The proportion of Tregs within the CD4^+^ T‐cell compartment was then calculated and incorporated into the present dataset for subsequent survival analysis.

### 2.19 Multiplex immunohistochemistry (mIHC)

FFPE tumour tissue sections (4‐µm thick) from 13 patients at the CHCAMS were used for mIHC. The multiplex immunofluorescence (MIF) staining kit was purchased from Nanjing Freethinking Biotechnology Co. Ltd. After deparaffinization in xylene, rehydration through a graded ethanol series and washing, antigen retrieval was performed by boiling the sections in Tris‐EDTA buffer at 95°C. Endogenous peroxidase activity was blocked by incubation with hydrogen peroxide for 10 min. Staining was performed using the Think color fluorescent mIHC kit (FreeThinking Biosciences, Nanjing, China) combined with a fluorophore‐conjugated tyramide signal amplification (TSA) kit (FreeThinking Biosciences, Nanjing, China) for signal enhancement. Sequential rounds of primary antibody incubation, secondary antibody incubation and TSA development were conducted, with epitope retrieval and protein blocking repeated between each round. Nuclei were counterstained with DAPI. Whole‐slide images were acquired using a digital slide scanner (3DHISTECH) and viewed with the SlideViewer software (3DHISTECH). Quantitative analysis was performed using the HALO Highplex FL module (version 3.4.2986.257, Indica Labs) to determine the number of positively stained cells and their percentage relative to total nucleated cells. The primary antibodies included CD20 (48750, CST, 1:500), NFKB (ab51608, Abcam, 1:1000), FOXP3 (98377, CST, 1:500), CD4 (ab133616, Abcam, 1:1000), TNFRSF4 (ab264466, Abcam, 1:4000), STAT4 (ab284408, Abcam, 1:1200) and T‐bet (ab307193, Abcam, 1:1000).

### 2.20 Statistical analysis

All statistical analyses were performed using Python 3.10.12. Continuous variables are reported as medians with interquartile ranges (IQRs) and were compared using the Wilcoxon rank‐sum test. Categorical variables are summarized as frequencies (percentages), with intergroup differences assessed by Fisher's exact test. To mitigate potential confounding, a nearest 3:1 propensity score matching (PSM) was applied to balance baseline covariates (e.g., age, gender and aCCI) between KRAS‐mutant and KRAS‐wildtype groups. Univariate Cox regression was used to screen variables associated with OS and RFS. Variables with *p* < .05 in univariable analysis, together with clinically relevant factors (co‐mutation status and pathological stage) determined a priori, were entered into a multivariable Cox proportional hazards model. The Benjamini–Hochberg method was applied for multiple testing correction where appropriate. A two‐tailed *p*‐value <.05 defined statistical significance.

## 3 RESULTS

### 3.1 Clinical characteristics of resectable KRAS‐mutant and KRAS‐wildtype LUAD patients treated with NIT

**FIGURE 1:**
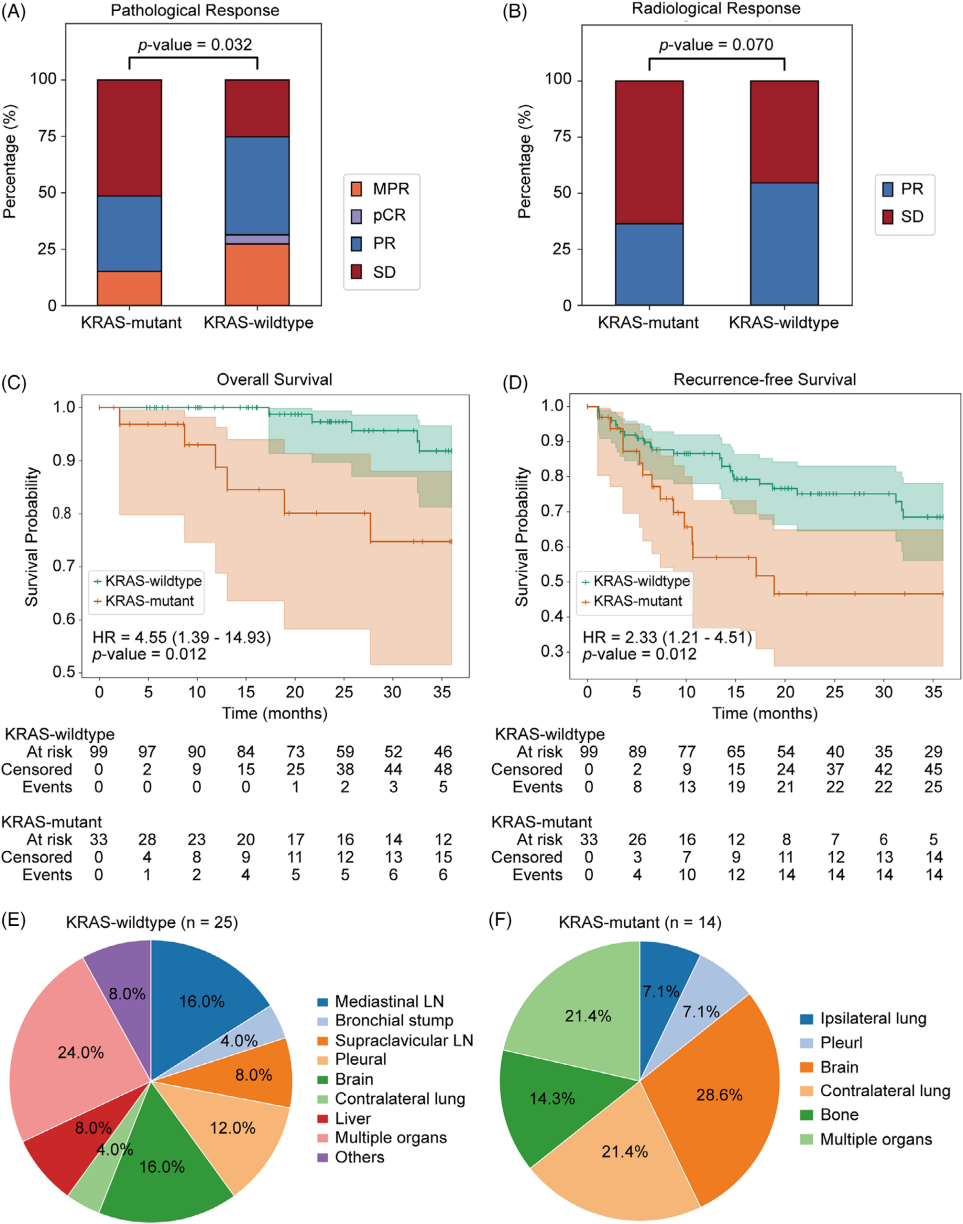
Pathologic and clinical outcomes of patients between Kirsten rat sarcoma viral oncogene homolog (KRAS)‐mutant and KRAS‐wildtype LUAD treated with neoadjuvant immunotherapy (NIT). (A) Pathological response for patients with KRAS‐wildtype versus KRAS‐mutant. (B) Radiological response for patients with KRAS‐wildtype versus KRAS‐mutant. Overall survival (C) and recurrence‐free survival (D) for patients with KRAS‐wildtype versus KRAS‐mutant. The recurrence pattern of the KRAS‐wildtype (E) and KRAS‐mutant groups (F). CR, complete response; MPR, major pathologic response; pCR, pathologic complete response; PR, partial response; SD, stable disease.

A total of 143 patients with clinical stage II‐III LUAD who received NIT were enrolled. This cohort comprised 106 KRAS‐wildtype and 37 KRAS‐mutant patients (Table S1). Following 3:1 PSM, 99 KRAS‐wildtype and 33 KRAS‐mutant patients were retained; baseline characteristics were well‐balanced between the two matched groups. Pathological and radiological responses for the KRAS‐mutant and KRAS‐wildtype groups are shown in Figure 1A,B. Radiologic evaluation indicated a PR rate of 36.4% (12/33) in the KRAS‐mutant group versus 54.5% (54/99) in the KRAS‐wildtype group (*p* = .070). Pathological response assessments further revealed that KRAS‐mutant patients exhibited significantly poorer pathological responses compared with KRAS‐wildtype patients (*p* = .032). In addition, perioperative outcomes were comparable between the two groups (Table S2).

### 3.2 Survival analysis between KRAS‐mutant and KRAS‐wildtype LUAD patients

With a median follow‐up of 42.2 months in the KRAS‐wildtype group and 37.3 months in the KRAS‐mutant group, survival analysis demonstrated significantly inferior outcomes in the KRAS‐mutant cohort. The 3‐year OS rate was 81.8% in KRAS‐mutant patients versus 94.9% in wildtype patients (HR  =  4.55, 95% CI: 1.39–14.93, *p* =  .012). Similarly, the 3‐year RFS rate was 57.6% in the KRAS‐mutant group compared with 74.7% in the KRAS‐wildtype group (HR =  2.33, 95% CI: 1.21–4.51, *p* =  .012) (Figure 1C,D). Univariate analysis identified KRAS‐mutant, sleeve resection and pneumonectomy as factors significantly associated with an increased risk of OS. KRAS mutation, pneumonectomy, pleural invasion, number of lymph node metastases and number of N2 lymph node metastases were risk factors for worse RFS. Crucially, our multivariate analysis confirmed that KRAS mutation status remained a significant independent predictor of poor OS and RFS (Table S3).

### 3.3 Recurrence patterns between KRAS‐mutant and KRAS‐wildtype groups

Recurrence patterns are summarized in Figure 1E,F. LR rates did not differ significantly between the KRAS‐mutant and KRAS‐wildtype groups (2.7% vs. 4.7%, *p* =  .960). In contrast, the rate of DR was significantly higher in the KRAS‐mutant group than in the KRAS‐wildtype group (39.1% vs. 18.9%, *p* =  .043) (Table S4).

### 3.4 Distinct immune microenvironments characterize KRAS‐mutant and KRAS‐wildtype LUAD

**FIGURE 2:**
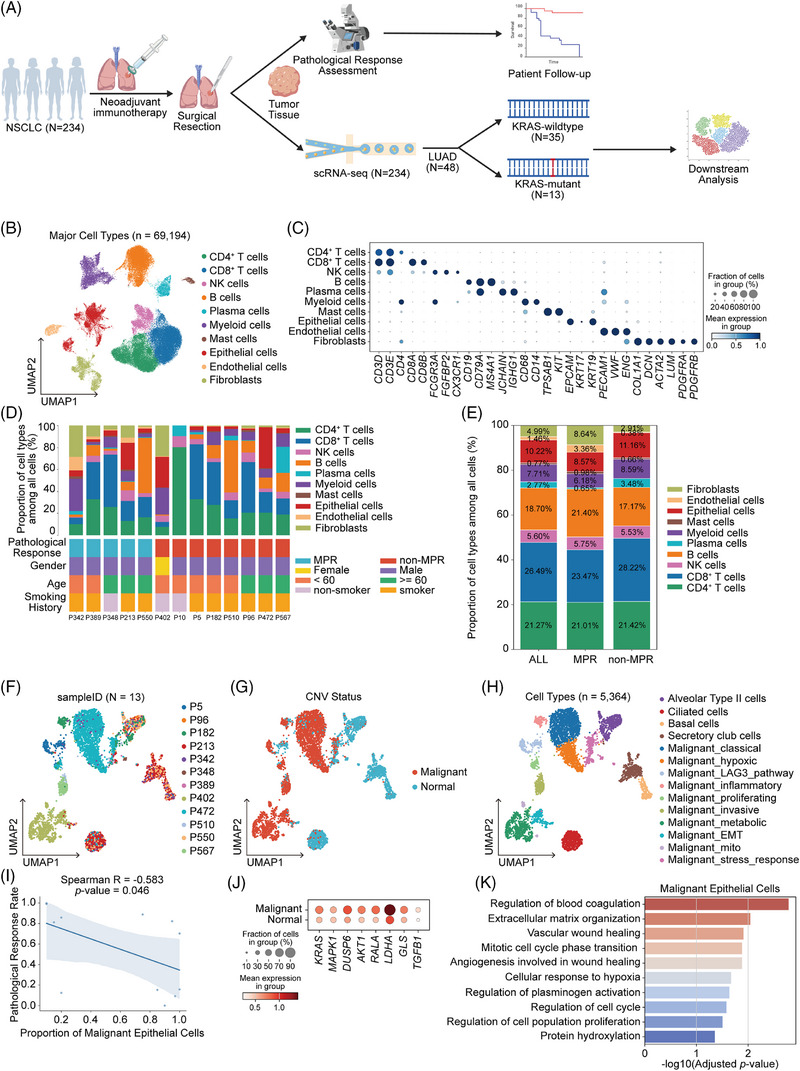
Malignant epithelial cell burden serves as a critical determinant of pathological response during neoadjuvant immunotherapy. (A) Schematic representation of the study design. (B) Uniform manifold approximation and projection (UMAP) plot of high‐quality cells (*n* = 69 194) from 13 Kirsten rat sarcoma viral oncogene homolog (KRAS)‐mutant lung adenocarcinoma (LUAD) patients, annotated by major cell types. (C) Dot plot showing the expression of representative markers for each major cell type. (D) Summary of the proportions of the major cell types among all cells and key clinical features for each KRAS‐mutant LUAD patient. (E) Distribution of the major cell types among pathological responses. (F–H) UMAP plot of 5364 epithelial cells after filtering, annotated by sample IDs (F), copy number variation (CNV) status (G) and cell types (H). (I) Spearman correlation between the proportion of malignant epithelial cells and pathological response rate. *n* = 13. (J) Dot plot showing the expression of key KRAS‐associated effector genes for epithelial cells. (K) Selected significant enrichment of differentially expressed genes (DEGs) upregulated in malignant epithelial cells in gene ontology terms. MPR, major pathologic response; NSCLC, non‐small cell lung cancer; scRNA‐seq, single‐cell RNA sequencing.

To characterize the heterogeneity of the TME following NIT, we performed scRNA‐seq on tumour specimens from 234 real‐world NSCLC patients (Figure 2A and Figure S1A,B). Within this cohort, 48 patients with LUAD were identified. We further refined our analysis to a subset of 306 825 cells, stratified by KRAS status: 13 patients harbouring KRAS mutations and 35 with the wildtype genotype (Figure 2A and Figure S1C). Although the global distribution of major cell types remained largely consistent across individual LUAD patients (Figure S1D), a detailed comparison revealed divergences between KRAS‐wildtype and KRAS‐mutant groups (Figure S1E). The functional states of tumour‐infiltrating lymphocytes also differed by genotype. CD8^+^ T cells in KRAS‐mutant tumours displayed a significantly higher exhaustion score relative to KRAS‐wildtype tumours (Figure S1F), indicating a more dysfunctional effector T‐cell phenotype associated with KRAS mutation. In addition, among CD4^+^ T cells, a pronounced increase in Treg signature scores was observed in KRAS‐mutant LUAD specimens (Figure S1G). These findings indicated that KRAS‐mutant was associated with a more robustly immunosuppressive microenvironment, potentially limiting the efficacy of neoadjuvant interventions.

### 3.5 Subgroup analysis of KRAS‐mutant LUAD patients

To further validate the predictive value of pathological response for survival, we conducted a subgroup analysis specifically within the KRAS‐mutant patient cohort. The survival results demonstrated a favourable trend in both RFS and OS for patients who achieved an MPR (*n* = 5) compared to those who did not (non‐MPR, *n* = 28). This result suggested that, even among KRAS‐mutant patients who have a poorer prognosis, a deep pathological response to NIT remains associated with superior long‐term survival outcomes (Figure S2). We also compared treatment responses and survival outcomes across different KRAS‐mutant subgroups. Co‐mutations identified in our KRAS‐mutant cohort included STK11 (*n* = 3), TP53 (*n* = 7) and no KEAP1 co‐mutations. Patients with KRAS–STK11 co‐mutations did not show a significant difference in pathological response compared to those with KRAS‐mutant alone, but they exhibited inferior OS (Figure S2). In contrast, no significant differences in treatment response or survival outcomes were observed between the KRAS‐mutant alone and KRAS‐TP53 co‐mutant groups (Figure S3). Similarly, when comparing KRAS G12C and KRAS non‐G12C variants, neither treatment response nor survival outcomes differed significantly between the two subgroups (Figure S4).

### 3.6 Distinct epithelial cell landscapes reflect divergent pathological responses after neoadjuvant immunotherapy

To further delineate the immune landscape associated with KRAS mutations, we constructed a single‐cell transcriptional atlas from 13 KRAS‐mutant LUAD tumours (5 MPR and 8 non‐MPR cases; Figure 2A and Table S5). Following stringent quality control to exclude low‐quality and doublet cells (criteria outlined in Figure S5A,B), a final dataset of 69 194 high‐quality cells was obtained from the 13 KRAS‐mutant LUAD patients (Figure 2B). These encompassed major lineages, including CD4^+^ and CD8^+^ T cells, NK cells, B cells, plasma cells, myeloid cells, mast cells, epithelial cells, endothelial cells and fibroblasts (Figure 2B,C, Figure S5C). Their distribution across clinicopathological categories is summarized in Figure 2D. Notably, non‐MPR tumours exhibited a higher fraction of epithelial cells compared with MPR tumours (Figure 2E), indicating that non‐MPR tumours were dominated by residual malignant components and a less immune‐infiltrated microenvironment.

Single‐cell profiling revealed pronounced inter‐patient heterogeneity within the epithelial compartment (Figure 2F). To further resolve these populations, we performed CNV inference using fibroblasts as reference cells to distinguish malignant from non‐malignant epithelial cells (Figure 2G and Figure S5D,E). We further clustered and annotated malignant and normal epithelial cells based on the gene expression profiles of individual epithelial cells (Figure 2H, Figure S5F and Table S6). Notably, the fraction of malignant epithelial cells was consistently higher in non‐MPR tumours (Figure S5G), and their abundance inversely correlated with pathological response rate (Figure 2I). Gene expression profiling showed that malignant epithelial cells significantly upregulated multiple KRAS effector genes compared to their non‐malignant counterparts (Figure 2J). Differential expression and functional enrichment analyses further indicated marked enrichment in pathways involving extracellular matrix (ECM) reorganization, dysregulated coagulation, hypoxia response and cell‐cycle progression (Figure 2K)—a profile consistent with an aggressive, therapy‐resistant epithelial phenotype. These pathway signatures delineate a TME conducive to immune evasion. Specifically, hypoxia stabilizes TFs like HIF‐1α, which can directly upregulate immunosuppressive molecules and recruit Tregs.^25^, ^26^ Concurrently, active ECM reorganization fosters fibrosis and stromal stiffening, creating a physical barrier that impedes the infiltration of cytotoxic T cells while also activating pro‐survival signalling in tumour cells.^27^, ^28^ Thus, the persistent malignant epithelial cells are not merely a marker of treatment resistance but may actively sculpt an immunosuppressive niche, potentially explaining the observed enrichment of suppressive Tregs and paucity of effector lymphocytes in non‐responding KRAS‐mutant tumours. These findings suggested that residual malignant epithelial burden reflects insufficient therapeutic efficacy and may serve as a cellular indicator of treatment resistance.

### 3.7 CD4T_Treg_TNFRSF4 cells define an immunosuppressive microenvironment associated with poor pathological response

**FIGURE 3:**
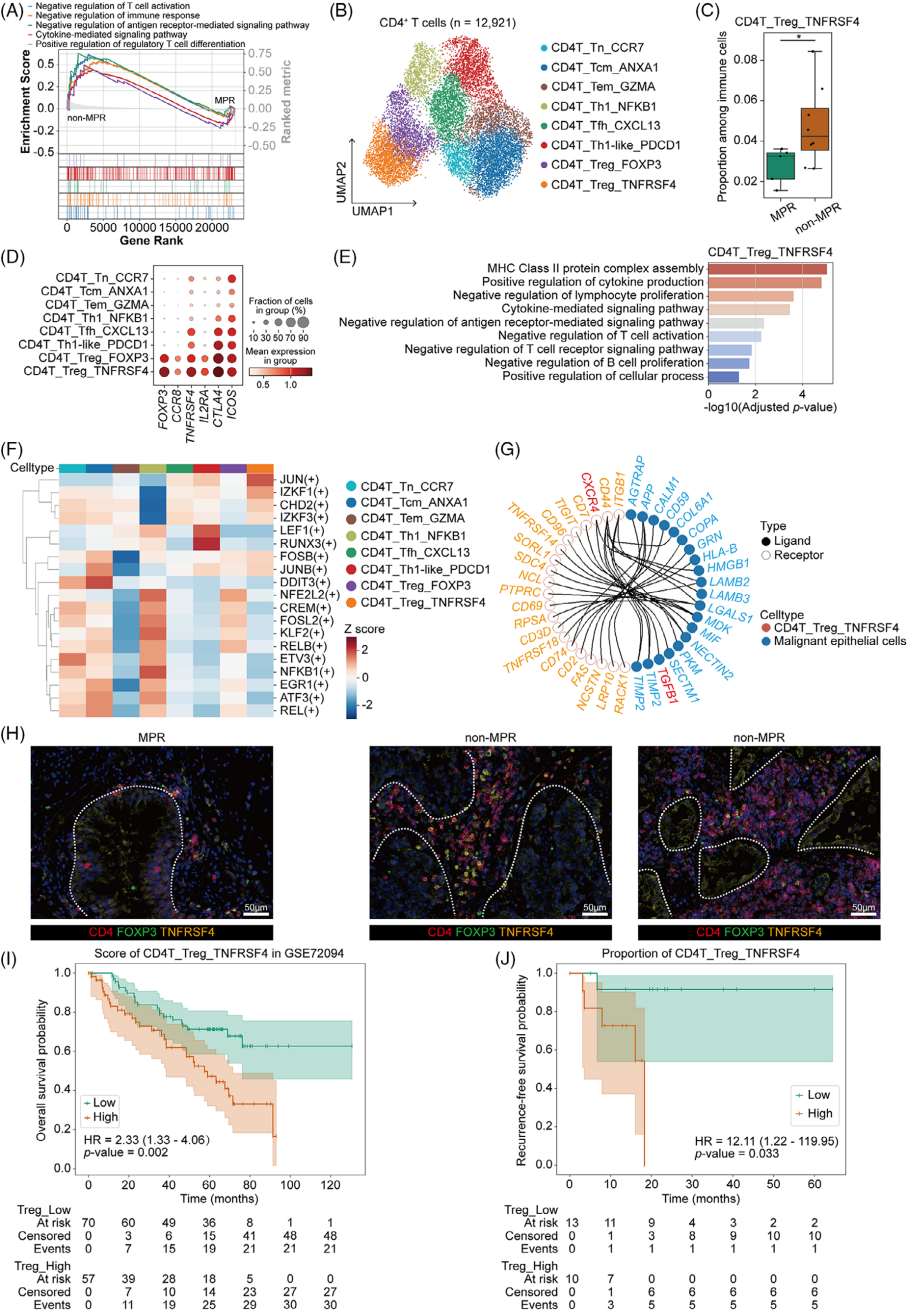
CD4T_Treg_TNFRSF4 cells as potential pathological response predictor. (A) Gene set enrichment analysis (GSEA) showing the enrichment scores for key gene ontology (GO) terms in T cells from non‐major pathological response (MPR) patients compared to MPR patients. (B) Uniform manifold approximation and projection (UMAP) plot of 12 921 CD4^+^ T cells, annotated by cell type subsets. (C) Boxplot showing the proportion of CD4T_Treg_TNFRSF4 subset among all immune cells for the MPR group and the non‐MPR group. MPR group, *n* = 5; non‐MPR group, *n* = 8. Mann–Whitney *U*‐test. *p < .05. Centre line, median; box limits, upper and lower quartiles; whiskers, 1.5× interquartile range. (D) Dot plot showing the expression of key Tregs‐associated genes for CD4^+^ T cells. (E) Selected significant enrichment of DEGs upregulated in CD4T_Treg_TNFRSF4 in Gene Ontology terms. (F) Heatmap showing the average *Z*‐scaled activity scores of transcription factors across CD4^+^ T‐cell subsets. (G) Chord diagram depicting selected statistically significant ligand–receptor interactions (*p* < .05) between malignant epithelial cells and CD4T_Treg_TNFRSF4 cells, specifically those mediated by the top 20 ligands from malignant epithelial cells (ranked by interaction score). (H) Representative multiplex immunohistochemistry (mIHC) staining of CD4^+^FOXP3^+^TNFRSF4^+^ Tregs in MPR and non‐MPR samples. (I) Kaplan–Meier curve showing the overall survival of patients categorized by the score of CD4T_Treg_TNFRSF4 in LUAD patients in the GSE50081 cohort. Log‐rank test. (J) Kaplan–Meier curve showing the recurrence‐free survival of patients categorized by the proportions of CD4T_Treg_TNFRSF4 among CD4^+^ T cells in the cohort combining patients in GSE280232 and in this study. Log‐rank test.

The persistence of a substantial proportion of malignant epithelial cells in non‐MPR patients following NIT and surgical resection suggested the presence of an immune‐resistant microenvironment incapable of mounting effective antitumour activity. In line with this observation, malignant epithelial cells showed increased expression of transforming growth factor‐beta (TGF‐β) (Figure 2J), a cytokine that promotes the differentiation of naïve T cells into Tregs and fosters an immunosuppressive niche.^29^ This observation prompted us to hypothesize that T‐cell dysfunction might play a pivotal role in the immunosuppressive TME. We thus performed GSEA on T‐cell subsets, which revealed that in the non‐MPR group, T cells exhibited significant enrichment of gene signatures related to cytokine‐mediated signalling pathways, accompanied by attenuated immune responses (Figure 3A). We further performed clustering and cell type annotation of CD4^+^ T cells and identified eight transcriptionally distinct subsets (Figure 3B, Figure S6A and Table S7). Indeed, one subset of Tregs, CD4T_Treg_TNFRSF4, was markedly enriched in the non‐MPR group (Figure 3C), suggesting its critical role in immune escape and resistance to immunotherapy.

Co‐expression of high levels of FOXP3, CCR8, IL2RA, CTLA4 and TNFRSF4 in Tregs reflects a potent immunosuppressive phenotype in NSCLC.^15^ Consistent with this, we revealed a pronounced upregulation of these key immunoregulatory molecules in CD4T_Treg_TNFRSF4 (Figure 3D). To further elucidate the functional state of this cell subset, we performed enrichment analysis on genes differentially expressed in the CD4T_Treg_TNFRSF4 subset. Results demonstrated significant enrichment of multiple immune‐inhibitory pathways and processes, including ‘negative regulation of T‐cell activation’, ‘negative regulation of lymphocyte proliferation’, ‘negative regulation of antigen receptor‐mediated signalling pathway’ and ‘negative regulation of T‐cell‐mediated immunity’ (Figure 3E). Additionally, terms such as ‘MHC class II protein complex assembly’ and ‘positive regulation of cytokine production’ suggested a role in modulating antigen presentation and cytokine‐mediated immune suppression (Figure 3E). TF activity analysis further demonstrated selective upregulation of IKZF1 and IKZF3‐stabilizers and functional enhancers of Tregs in CD4T_Treg_TNFRSF4 cells (Figure 3F). Ligand–receptor interaction analysis between epithelial cells and CD4T_Treg_TNFRSF4 revealed significant crosstalk via immunoregulatory pairs such as TGFβ1–CXCR4 (Figure 3G), highlighting epithelial–Treg communication as a potential mechanism for maintaining immune tolerance. Finally, we experimentally validated the proximity between Tregs and malignant epithelial cells, demonstrating that Tregs were significantly more enriched around tumour regions in non‐MPR patients (Figure 3H).

To further validate the clinical relevance of the identified CD4T_Treg_TNFRSF4 cells, we first turned to the bulk RNA‐seq data from the GSE50081^24^ cohort. We specifically selected LUAD samples to maintain genetic context relevance. By applying a gene expression signature representative of CD4T_Treg_TNFRSF4 to these samples, we computed a Treg abundance score for each patient. Subsequent Kaplan–Meier survival analysis demonstrated that this score served as a significant predictor of OS (*p* = .002) (Figure 3I). Next, we leveraged a publicly available KRAS‐mutant NSCLC cohort receiving NIT (GSE280232).^12^ Cells annotated as CD4T_Treg_TNFRSF4 were extracted from this validation cohort through re‐clustering of scRNA‐seq data (Figure S6B–G). After integrating these data with our own dataset, we stratified all 23 patients into high and low groups based on the relative frequency of CD4T_Treg_TNFRSF4 within the total CD4^+^ T‐cell population. Notably, Kaplan–Meier analysis revealed that a higher infiltration level of CD4T_Treg_TNFRSF4 significantly increased the risk of recurrence following NIT (*p* = .033) (Figure 3J).

### 3.8 Th1 cells are enriched in MPR tumours and transcriptionally associated with antitumour‐immune activation

**FIGURE 4:**
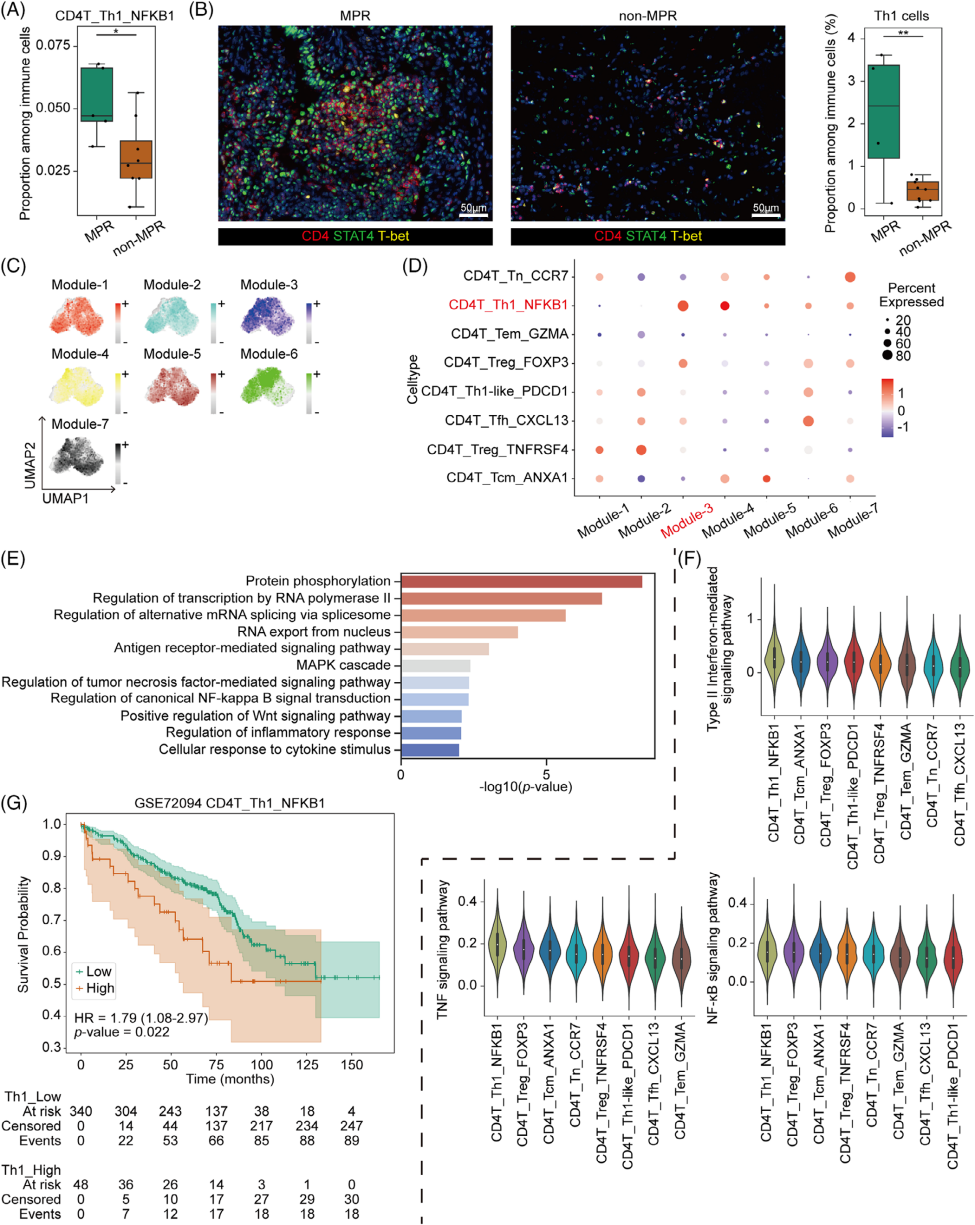
Th1 cells promote antitumour responses through multiple signal transduction pathways. (A) Boxplot showing the proportion of CD4T_Th1_NFKB1 subset among all CD4^+^ T cells for the major pathological response (MPR) group and non‐MPR group. MPR group, *n* = 5; non‐MPR group, *n* = 8. Mann–Whitney *U*‐test. *p < .05. Centre line, median; box limits, upper and lower quartiles; whiskers, 1.5× interquartile range. (B) Multiplex immunohistochemistry (mIHC) staining of CD4^+^STAT4^+^T‐bet^+^ Th1 cells in MPR and non‐MPR samples. Left, representative mIHC images of MPR and non‐MPR samples. Scale bar, 50 µm. Right, bar plot showing the cell proportion of CD4^+^STAT4^+^T‐bet^+^ Th1 cells. MPR group, *n* = 4; non‐MPR group, *n* = 9. *t*‐test. **p < .01. (C) Uniform manifold approximation and projection (UMAP) plots showing the scores of 7 gene modules among CD4^+^ T‐cell subtypes. (D) Dot plot showing the expression of seven gene modules for CD4^+^ T cells. Selected gene in Module 3 is indicated by a red label. (E) Selected significant enrichment of genes in Module 3 in gene ontology terms. (F) Violin plots showing the scores of the ‘type ii interferon‐mediated signalling pathway’ from the gene ontology (GO) database (top), ‘TNF signalling pathway’ from the Kyoto Encyclopedia of Genes and Genomes (KEGG) database (bottom left) and ‘NFKB signalling pathway’ from the KEGG database (bottom right) across CD4^+^ T‐cell subsets. White dot, median; thick black bar, interquartile range; thin vertical line, entire range excluding outliers. (G) Kaplan–Meier curves showing the overall survival of patients categorized by the score of CD4T_Th1_NFKB1 in patients in the GSE72094 cohort. Log‐rank test.

Th1 cells orchestrate immune responses by activating CD8^+^ T cells and NK cells, thereby potentiating cellular immunity.^30^ We successfully characterized Th1 cells in our scRNA‐seq data (Figure 3B) and revealed that the frequency of Th1 cells was significantly increased in the MPR group (Figure 4A). Through mIHC analysis, we experimentally validated that frequency of Th1 was significantly higher in MPR patients (Figure 4B). In order to identify the key molecular features of Th1 cells, we performed hdWGCNA within CD4^+^ T cells. During the construction of the co‐expression network (Figure S7A,B), we determined that a soft threshold power of *β* = 6 achieved optimal connectivity when the scale‐free topology fit index reached .865. Seven distinct gene modules were identified in total (Figure S7C), with their interrelationships summarized in Figure S7D. We assessed the module scores for each CD4^+^ T‐cell subset and found that Module 3 was relatively highly expressed in Th1 cells (Figure 4C,D). We further calculated modular connectivity to determine the connectivity of each gene based on the characterized genes (Figure S7E and Table S8).

By performing enrichment analysis on genes in Module 3, we revealed that Th1 cells were transcriptionally linked to diverse processes critical for antitumour immunity through involvement in transcriptional regulation, signalling transduction and post‐transcriptional processing (Figure 4E). Key enriched pathways include RNA polymerase II‐mediated transcription, alternative splicing via the spliceosome and protein phosphorylation, indicating robust activity in genetic information flow and response modulation. Th1 cells also exhibit activation in essential immune‐related cascades such as the MAPK cascade, Wnt signalling, NFKB transduction and TNF‐mediated signalling pathway, underscoring their central role in regulating inflammatory and immune responses. Moreover, we calculated the score of several signalling pathways in CD4^+^ T cells. Notably, Th1 cells exhibited the highest mean scores across INF‐γ, TNF and NFKB signalling pathways (Figure 4F), corresponding to the findings supporting the antitumour function of Th1 cells.^31^, ^32^, ^33^ Importantly, by analysing bulk RNA sequencing data from the GSE72094 cohort, we confirmed that a high infiltration score of Th1 cells was significantly associated with improved OS (Figure 4G). Collectively, these findings suggest the enrichment of Th1 cells serves as a hallmark of a favourable therapeutic response and may reflect a transcriptionally active, immune‐supportive TME in KRAS‐mutant NSCLC.

### 3.9 Identification of a progenitor‐exhausted B‐cell state linked to pathological MPR and enhanced antitumour immunity

**FIGURE 5:**
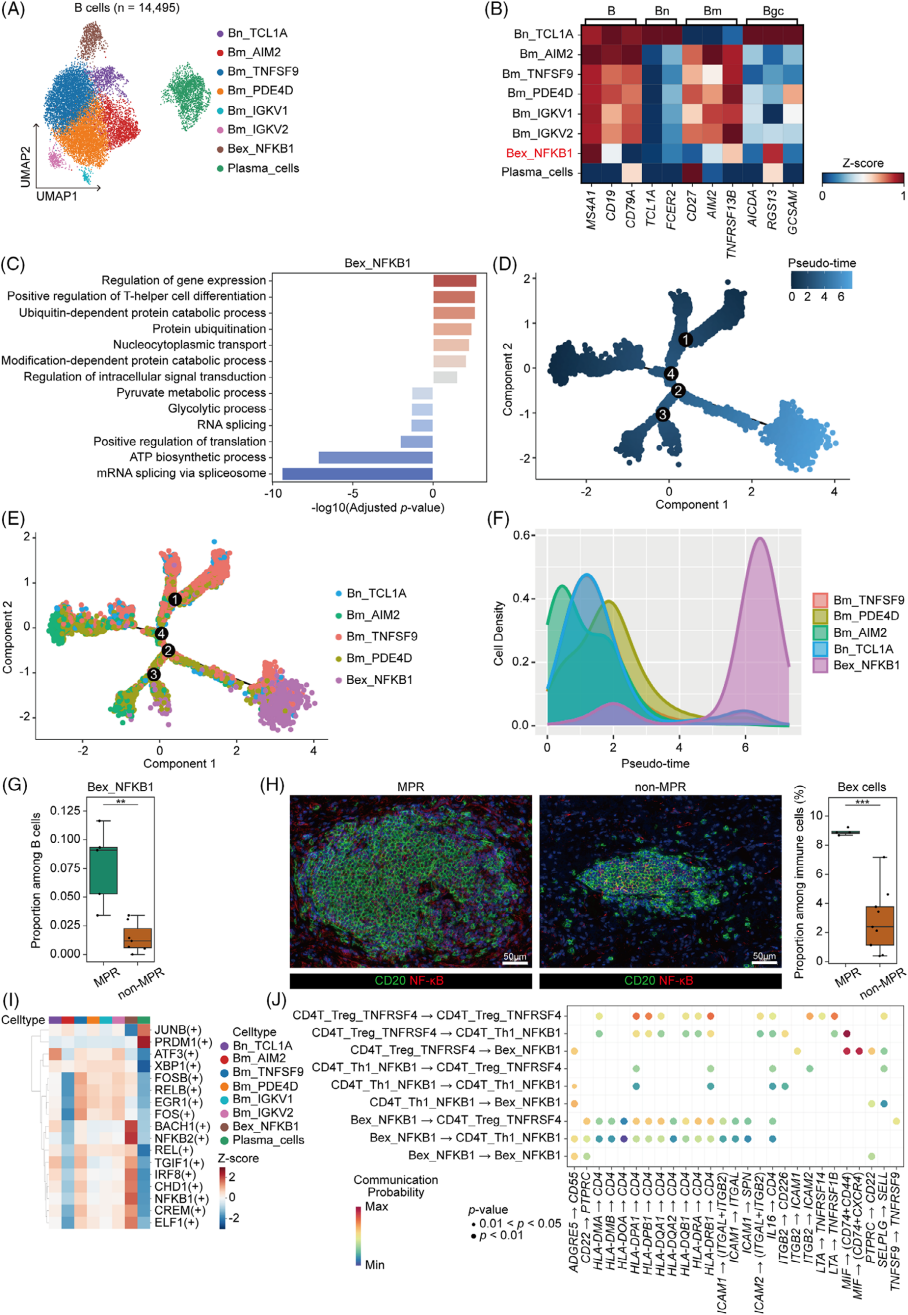
Identification of a progenitor‐exhausted B‐cell state linked to pathological major pathological response (MPR) and enhanced antitumour immunity. (A) Uniform manifold approximation and projection (UMAP) plot of 14 495 B cells, annotated by cell type subsets. (B) Heatmap showing the expression of markers of B cells (B), naïve B cells (Bn), memory B cells (Bm) and germinal centre B cells (Bgc) among B‐cell subtypes. (C) Selected significant enrichment of DEGs upregulated (right) or downregulated (left) in Bex_NFKB1 in gene ontology terms. (D–E) Predicted pseudo‐time trajectory of B‐cell subsets. (F) Cell density of B‐cell subsets along pseudo‐time. (G) Boxplot showing the proportion of Bex_NFKB1 subset among all B cells for the MPR group and the non‐MPR group. MPR group, *n* = 5; non‐MPR group, *n* = 8. Mann–Whitney *U*‐test. *p < .05. Centre line, median; box limits, upper and lower quartiles; whiskers, 1.5× interquartile range. (H) Multiplex immunohistochemistry (mIHC) staining of CD20^+^NF‐KB^+^ Bex cells in MPR and non‐MPR samples. Left, representative mIHC images of MPR and non‐MPR samples. Scale bar, 50 µm. Right, bar plot showing the cell proportion of CD20^+^NF‐KB^+^ Bex cells. MPR group, *n* = 4; non‐MPR group, *n* = 9. *t*‐test. ***p < .001. (I) Heatmap showing the average *Z*‐scaled activity scores of transcription factors across B‐cell subsets. (J) Dot plot showing the details of predicted ligand–receptor interactions between CD4T_Treg_TNFRSF4, CD4T_Th1_NFKB1 and Bex_NFKB1.

Building on the central role of CD4^+^ T cells and Th1‐mediated immunity outlined above, we further sought to determine the contribution of B cells, another pivotal component of the adaptive immune system, to the antitumour microenvironment. Clustering and cell type annotation of B cells resulted in eight cell subsets (Figure 5A and Table S9). Interestingly, we identified a distinct B‐cell subset (Bex_NFKB1) which, while expressing canonical B‐cell markers, could not be classified as naïve B cells (Bn), memory B cells (Bm) or germinal centre B cells (Bgc) (Figure 5B). This unique phenotype led us to propose this cluster as a previously unrecognized B‐cell subset. Notably, this unique subset expressed high levels of CD20 but reduced CD19 and CD79A compared with conventional B cells (Figure 5B). Based on this atypical expression profile, we hypothesized that these cells may represent an exhausted B‐cell population, which we designated as Bex (exhausted B cells). Subsequent enrichment analysis confirmed hallmark features of exhaustion in Bex (Figure 5C), including suppression of B‐cell receptor signalling, mRNA translation and ATP biosynthesis, along with upregulation of stress‐response pathways such as protein degradation, DNA damage response and programmed necrosis. Moreover, analysis of metabolic activities revealed that Bex exhibited a distinct metabolic signature, characterized by significant enrichment in pathways related to stress adaptation and survival maintenance (Figure S8A).^34^, ^35^

Considering the terminal differentiation properties of exhausted B cells, we next explored the lineage relationships and differentiation trajectories among B‐cell subsets. To ensure a robust trajectory inference, we excluded cell subsets that showed no evident developmental connectivity on the UMAP visualization (Figure 5A) and re‐computed differential genes across the remaining subsets for subsequent Monocle2 analysis (Table S10). Using this computational approach, we further elucidated the potential developmental trajectory of B‐cell subsets and confirmed that Bex resided at the terminal ends of differentiation paths (Figure 5D–F). Similar results were observed using other trajectory analysis tools like Slingshot (Figure S8B) and CytoTRACE (Figure S8C). Although Bex has previously been implicated in autoimmune diseases,^36^ in our study, we observed that the frequency of Bex was significantly higher in the MPR group (Figure 5G), implying that this population may emerge in the context of effective antitumour‐immune activation. Through mIHC analysis, we experimentally validated that frequency of Bex was significantly higher in MPR patients (Figure 5H). Using TF activity analysis, we indicated the co‐activation of several key TFs in Bex (Figure 5I), suggesting a distinctive regulatory architecture underlying the Bex phenotype.

Interestingly, we observed a strong positive correlation between the frequencies of Bex and Th1 cells (Figure S8D). Based on bioinformatics analysis, including ligand–receptor interaction analysis, it further revealed extensive communication between these two subsets (Figure S8E). Therefore, we further performed CellChat analysis to delve into the interactions among Tregs, Th1 cells and Bex in the TME, which revealed an extensive cellular crosstalk between these cell subsets (Figure 5J). For example, the cognate interaction between HLA class II molecules on Bex cells and CD4 on Th1 cells (e.g., HLA‐DRB1‐CD4) suggested that Bex might function as antigen‐presenting cells to directly prime and activate tumour antigen‐specific Th1 cells. In contrast, Treg‐derived MIF signalling through the MIF‐CD74 axis on Bex and Th1 cells might contribute to immune suppression and reduced effector function.^37^ Altogether, these results illustrated a potentially dynamic regulatory network where Bex cells may enhance antitumour immunity through Th1 engagement, whereas Tregs may counterbalance this effect, underscoring the complex immune equilibrium that shapes therapeutic outcomes following NIT.

## 4 DISCUSSION

This retrospective study evaluated treatment responses and survival outcomes in patients with clinical stage II‐III LUAD receiving NIT, comparing KRAS‐mutant and KRAS‐wildtype subgroups. The KRAS‐mutant cohort displayed significantly poorer pathological responses and inferior long‐term survival. Multivariate Cox regression confirmed KRAS mutation as an independent adverse prognostic factor for both overall and RFS. The relatively poor response to NIT in KRAS‐mutant LUAD may be attributed to TME characteristics. KRAS‐mutant tumours frequently exhibit an ‘immune‐cold’ phenotype, characterized by diminished CD8^+^ T‐cell infiltration, low PD‐L1 expression, and elevated levels of immunosuppressive cytokines, including VEGF and TGF‐β.^38^ Certain co‐mutation patterns, such as STK11 or KEAP1,^39^ may further exacerbate genomic instability and foster an immunosuppressive microenvironment. In addition, constitutive KRAS signalling can impair interferon pathways and antigen presentation machinery, hindering effective immune recognition.^40^

This study also found that the KRAS‐mutant group had a higher incidence of DR. These findings suggested that KRAS‐mutant LUAD may possess distinct metastatic biological properties, including a potential predisposition for brain metastasis, possibly mediated through RANKL/PI3K‐AKT pathway activation.^38^ A previous study has indicated that high RANKL expression not only suppresses PD‐L1 expression but also impairs the secretion of CXCL9/10/11 by macrophages, leading to reduced CD8^+^ T‐cell infiltration and ultimately promoting metastasis.^38^ Furthermore, KRAS G12C mutation activates glycolysis via mTOR signalling and SLC4A7‐mediated lactate efflux, potentially contributing to the immunosuppressive microenvironment and facilitating distant metastasis.^39^ Under the selective pressure of NIT, KRAS‐mutant tumours may accelerate clonal evolution, giving rise to immune‐resistant subclones that contribute to DR.^12^ These results emphasized that patients with KRAS‐mutant LUAD may require intensified postoperative adjuvant therapy and close follow‐up surveillance, including regular neuroimaging for early detection of brain metastases, even after receiving neoadjuvant therapy and undergoing surgery. Therefore, we propose that for KRAS‐mutant patients, post‐operative management should be intensified. This could include (1) optimized adjuvant therapy: incorporating KRAS inhibitors into the adjuvant setting for G12C–mutant patients who do not achieve pCR, similar to the ADAURA paradigm for EGFR^41^; and (2) enhanced surveillance: implementing routine contrast‐enhanced brain MRI and high‐sensitivity ctDNA monitoring every 3–6 months post‐surgery to detect distant metastases early.

Although our findings confirmed that KRAS‐mutant status was an independent predictor of poor survival, the identification of distinct immune subsets offers actionable targets to reverse this resistance. Specifically, the enrichment of CD4T_Treg_TNFRSF4 in non‐responders suggested that these cells are not merely bystanders but active drivers of immune tolerance. Although TNFRSF4 was traditionally targeted by agonists to boost effector T cells, a previous study revealed its high co‐expression with CCR8 and CTLA4 on intratumoural Tregs.^15^ This distinction is critical for clinical translation: Rather than simple agonism, therapeutic strategies should prioritize Treg‐depleting mechanisms. Emerging agents such as anti‐CCR8 antibodies or ADCC‐optimized anti‐CTLA‐4 antibodies (e.g., ipilimumab) could selectively eliminate this immunosuppressive population without impairing peripheral immunity, thereby unleashing the antitumour activity of Th1 cells.^42^, ^43^

For the substantial proportion of patients with KRAS G12C mutations, the integration of G12C‐specific inhibitors (e.g., sotorasib or adagrasib) with NIT represents a promising frontier. KRAS inhibition has been shown to increase T‐cell infiltration and MHC‐I expression, essentially turning ‘cold’ tumours ‘hot’. Early data from trials suggested that combining adagrasib with pembrolizumab shows manageable safety and encouraging efficacy.^8^ Our study supports this rationale, suggesting that reducing the malignant epithelial burden via targeted therapy could alleviate the hypoxic and immunosuppressive signals (e.g., TGF‐β) that drive Treg differentiation, thereby creating a window for PD‐1 inhibitors to function. To move towards precise clinical management, the specific immune cell subsets identified here could serve as predictive biomarkers. We proposed that quantification of CD4T_Treg_TNFRSF4 infiltration and the Bex/Th1 cell ratio in pre‐treatment biopsy specimens could guide therapeutic decision‐making. Patients with a ‘Bex/Th1‐dominant’ profile may be ideal candidates for standard chemoimmunotherapy. In contrast, patients with a ‘Treg‐dominant’ profile might benefit from an intensified ‘dual‐immunotherapy’ approach (PD‐1(+) CTLA‐4 blockade) or the addition of a KRAS inhibitor to the neoadjuvant regimen. Additionally, therapeutic agents that enhance B‐cell antigen presentation, such as CD40 agonists, could be explored to boost the functionality of Bex cells, amplifying the synergistic Bex–Th1 loop.^44^, ^45^

In addition, our findings highlight a unique tumour‐immune ecosystem in KRAS‐mutant LUAD receiving NIT. We observed that a higher burden of malignant epithelial cells correlated with poor pathological response, accompanied by proliferative, metabolic and stromal remodelling programmes. In contrast, tumours achieving MPR were characterized by a strong pro‐inflammatory axis centred on Th1 cells. These cells exhibited canonical IFNG‐driven signatures and occupied network hubs in our ligand–receptor analysis. Although our data support an association between Th1 abundance and enhanced antitumour activity, functional consequences, such as CD8^+^ T cell or NK cell activation,^46^ remain inferred rather than directly demonstrated. The inverse relationship between Th1 activity and Treg infiltration underscores a dynamic balance in the TME, in which immune clearance may predominate under Th1 dominance and tolerance under Treg prevalence. This paradigm aligns with clinical evidence linking Th1‐associated cytokines to better immunotherapy outcomes,^32^, ^33^ and interventions capable of tipping this balance may facilitate therapeutic efficacy.

Perhaps the most unanticipated observation was the emergence of a distinct B‐cell state, which we term Bex. Unlike classical exhausted lymphocytes, Bex retained antigen‐presenting capacity and were specifically enriched in responders. Their strong correlation with Th1 abundance, coupled with predicted HLA class II–CD4 interactions, suggested that Bex acted not as passive bystanders but as facilitators of Th1 polarization and maintenance. This dual identity challenges the conventional notion that exhaustion is synonymous with irreversible dysfunction. Instead, Bex may represent a ‘productive exhaustion’ state, characterized by adaptation to chronic antigen stimulation while retaining the capacity to reinforce antitumour immunity. This recognition expands the spectrum of B‐cell heterogeneity in cancer and highlights Bex as a previously overlooked player in the tumour‐immune ecosystem, which may open new therapeutic avenues for KRAS‐mutant LUAD. Our study elucidated a potential mechanism by which KRAS mutations orchestrate immune resistance. We propose that the constitutive activation of KRAS functions as an upstream driver that rewires the tumour cell metabolism (downstream upregulation of glycolysis and hypoxia pathways). This metabolic stress, coupled with the direct downstream secretion of TGF‐β and VEGF, creates an exclusion niche.^11^ This niche promotes the recruitment and maintenance of CD4T_Treg_TNFRSF4 while suppressing effective Th1 responses. Thus, the ‘function’ of the KRAS mutant target extends beyond intrinsic tumour cell proliferation to the active engineering of an immunosuppressive ecosystem. Furthermore, Bex cells may function as ‘activated sentinels’, presenting antigens via HLA class II molecules and, through the expression of molecules such as ICOSL, receiving IFNγ signals from Th1 cells to amplify their own function. This establishes a positive feedback loop that collaboratively promotes antitumour immunity.^47^ In contrast, Tregs act as ‘multifaceted suppressors’, employing diverse mechanisms, including CTLA‐4‐mediated competitive inhibition, secretion of immunosuppressive cytokines and the CD39‐CD73‐adenosine pathway. Adenosine signalling via the A2A receptor can directly promote Treg proliferation and enhance their suppressive function while concurrently inhibiting the effector functions of CD8^+^ T cells.^48^ In addition, Treg‐derived MIF signalling through the MIF‐CD74 axis on Bex and Th1 cells may contribute to immune suppression and reduced effector function.^37^ Collectively, these results illustrate a potentially dynamic regulatory network wherein Bex cells may enhance antitumour immunity through engagement with Th1 cells, whereas Tregs counterbalance this effect, underscoring the complex immune equilibrium that shapes therapeutic outcomes following NIT.^37^ Nevertheless, further experimental verification was required to confirm the functional significance of this interplay.

Our findings also indicated that patients with KRAS–STK11 co‐mutations have worse OS compared to those with KRAS‐mutant alone. Previous studies have shown that although KRAS mutations, often found in smoking‐associated NSCLC, can increase TMB and immunogenicity, co‐occurrence with STK11 or KEAP1 significantly worsens prognosis. Patients with KRAS and STK11/KEAP1 co‐mutations exhibited worse response rates to PD‐1 inhibitors (ORR 7.4%–11.6%) and poor survival (median OS 6.4 months).^12^, ^49^ The OS for patients with KRAS and STK11/KEAP1 co‐mutations was significantly lower than for those with KRAS‐mutant alone (6.4 vs. 15.0 months). Potential mechanisms include STK11 altering metabolism via the AMPK/mTOR pathway, inhibiting the STING pathway and T‐cell activation, thereby fostering an immunosuppressive TME. Furthermore, KRAS–STK11 co‐mutations downregulate MHC class II and chemokine expression, impairing antigen presentation.^12^ However, recent evidence from the CheckMate‐227^50^ and POSEIDON trials^51^ suggested that combined anti‐PD‐1/CTLA‐4 therapy with chemotherapy shows superior efficacy compared to chemotherapy alone, particularly for patients with STK11/KEAP1 mutations (HR .62–.75). Prospective trials are warranted to further validate the potential of targeted strategies (e.g., the KRAS G12D inhibitor MRTX1133).^52^

Notably, KRAS G12C inhibitors extend beyond direct antitumour effects to include immunomodulation, restoring TME homeostasis by reversing KRAS‐driven immunosuppression and favouring antitumour immunity.^53^, ^54^ By suppressing Myc, KRAS G12C inhibition upregulates interferon signalling, thereby diminishing the infiltration of immunosuppressive cells while enhancing the recruitment, activation and antigen‐presenting capacity of cytotoxic T lymphocytes.^40^ This provided a key theoretical rationale for future exploration of combining KRAS inhibitors with ICIs in the neoadjuvant setting. Although NIT remains investigational in the neoadjuvant setting, preliminary data from advanced lung cancer provide both reference and caution. The combination of a KRAS G12C inhibitor (e.g., adagrasib) with pembrolizumab has shown encouraging efficacy in the first‐line treatment of advanced disease (with an ORR of approximately 71%). However, this regimen also carried a substantial toxicity burden, with grade ≥3 treatment‐related adverse events occurring in up to 39% of patients. Furthermore, resistance may emerge following KRAS inhibitor monotherapy, and whether combination immunotherapy can delay or overcome this resistance remains a major unknown.^55^, ^56^ Therefore, applying the combination of a KRAS inhibitor and NIT to operable LUAD urgently requires prospective clinical trials to systematically evaluate its safety (particularly perioperative complications), efficacy and optimal dosing sequence.

Several limitations should be noted. First, our analyses are based on single‐cell transcriptomics from post‐treatment resections, providing only cross‐sectional snapshots rather than dynamic trajectories of immune adaptation. The KRAS‐mutant subgroup remains relatively small, and functional causality of TGFB1‐Treg or Bex–Th1 interactions was not experimentally demonstrated. Second, although we utilized a large KRAS‐wildtype clinical cohort to establish the specificity of our findings to the KRAS‐mutant context, we did not employ KRAS‐knockout animal models or cell lines. Third, spatial resolution was not assessed, limiting our ability to confirm microanatomical niches of interaction. Future studies integrating spatial transcriptomics, functional perturbations and longitudinal sampling will be essential to establish causality and therapeutic potential. Fourth, although this study leveraged scRNA‐seq to deconvolute the TME, this approach captures gene expression signatures only at the mRNA level and does not directly reflect protein‐level expression differences. Future work should integrate technologies such as single‐cell proteomics and single‐cell ATAC‐seq to characterize cellular subpopulations from multi‐dimensional perspectives—transcriptomic, proteomic and epigenomic—thereby achieving a more comprehensive understanding of the TME.

In summary, KRAS‐mutant was associated not only with poorer pathological responses to NIT for LUAD patients but also serves as an independent prognostic biomarker for survival outcomes. In addition, our study characterized the immune microenvironments of KRAS‐mutant LUAD in which CD4T_Treg_TNFRSF4 is associated with suppressed antitumour immunity, whereas Th1 and Bex cells are linked to pro‐inflammatory networks and favourable pathological responses. These insights provide a rationale for combinatorial strategies: pairing PD‐1 blockade with KRAS pathway inhibition, TGF‐β/Treg‐targeted therapies (e.g., CCR8 or TNFRSF4 antagonism) or approaches that preserve Bex/Th1 functionality, with the potential to improve outcomes in KRAS‐mutant LUAD. However, the practical value of this strategy requires validation through future preclinical models and clinical trials.

## AUTHOR CONTRIBUTIONS

Sikai Wu and Jiheng Niu contributed substantially to the acquisition, analysis or interpretation of data, draft of the manuscript. Xiaowei Chen, Jinfei Li and Quanying Tang critically revised the manuscript. Zhenlin Yang and Shugeng Gao contributed substantially to the study conception and design. All authors interpreted the data and critically revised the manuscript. All authors read and approved the final manuscript.

## CONFLICT OF INTEREST STATEMENT

The authors declare no conflicts of interest.

## ETHICS STATEMENT

This study was conducted in accordance with the principles of the Declaration of Helsinki and its subsequent amendments. The study protocol was approved by the Institutional Review Board of CHCAMS (approval number: 22/492‐3694), which waived the requirement for individual informed consent for this retrospective analysis. All authors agree to the publication of this article.

## Supporting information



Supporting Information

Supporting Information

Supporting Information

Supporting Information

Supporting Information

Supporting Information

Supporting Information

Supporting Information

Supporting Information

Supporting Information

Supporting Information

Supporting Information

Supporting Information

Supporting Information

Supporting Information

## Data Availability

The data and code for analysis in this work are available to researchers upon reasonable request. Please e‐mail the corresponding author, Shugeng Gao, MD, at gaoshugeng@cicams.ac.cn.
