# Supplementary figures and images for: The landscape of responses to neoadjuvant immunotherapy in resectable Kirsten rat sarcoma viral oncogene homolog‐mutant lung adenocarcinoma: Clinical heterogeneity and correlative immunologic analysis
Source: Clin Transl Med. 2026 Apr 20;16(4):e70670. doi: 10.1002/ctm2.70670 (PMC13096693; doi:10.1002/ctm2.70670)

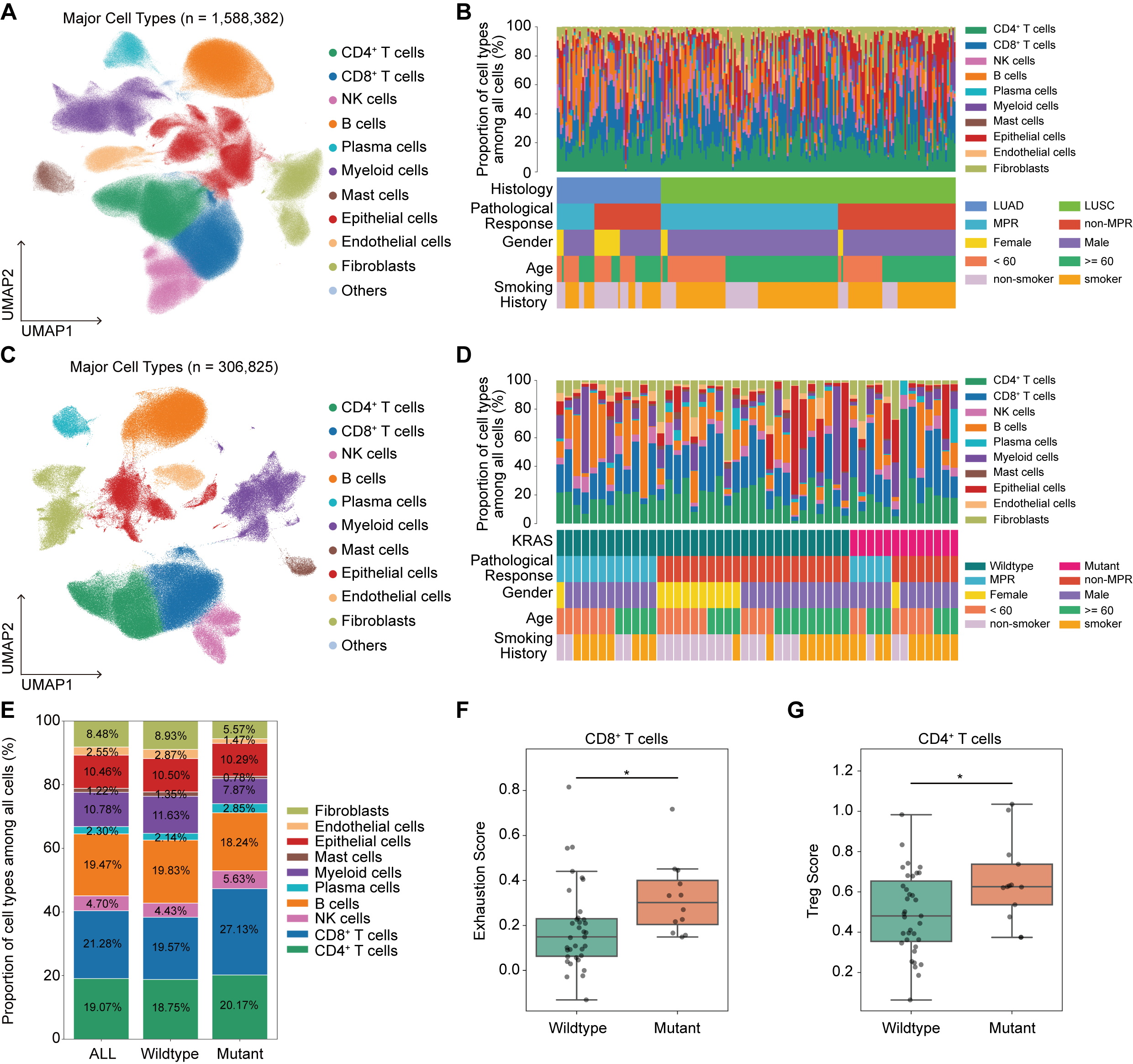

Supplement: Supplementary file 1 — Supporting Information [file CTM2-16-e70670-s006.tif]

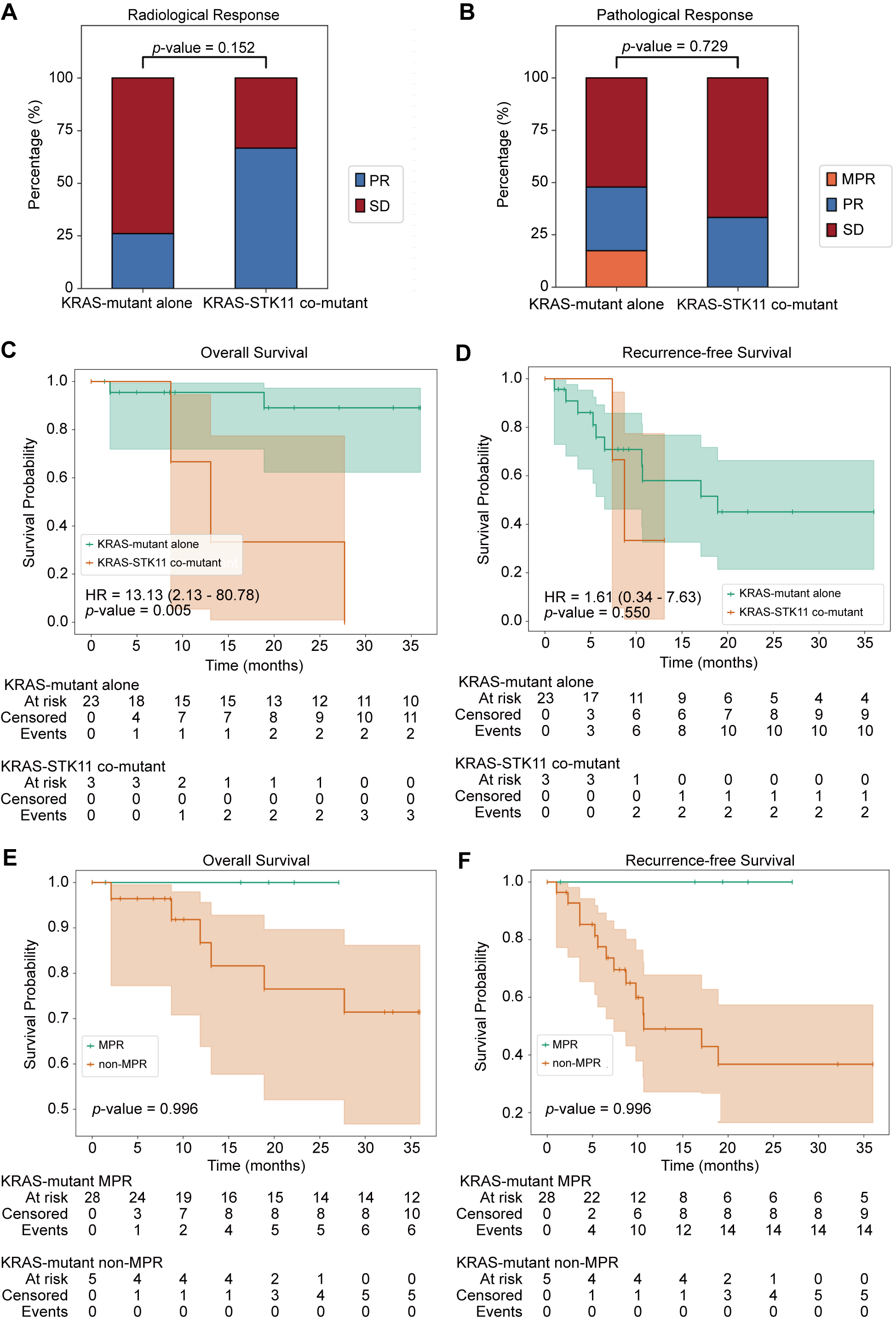

Supplement: Supplementary file 2 — Supporting Information [file CTM2-16-e70670-s007.tif]

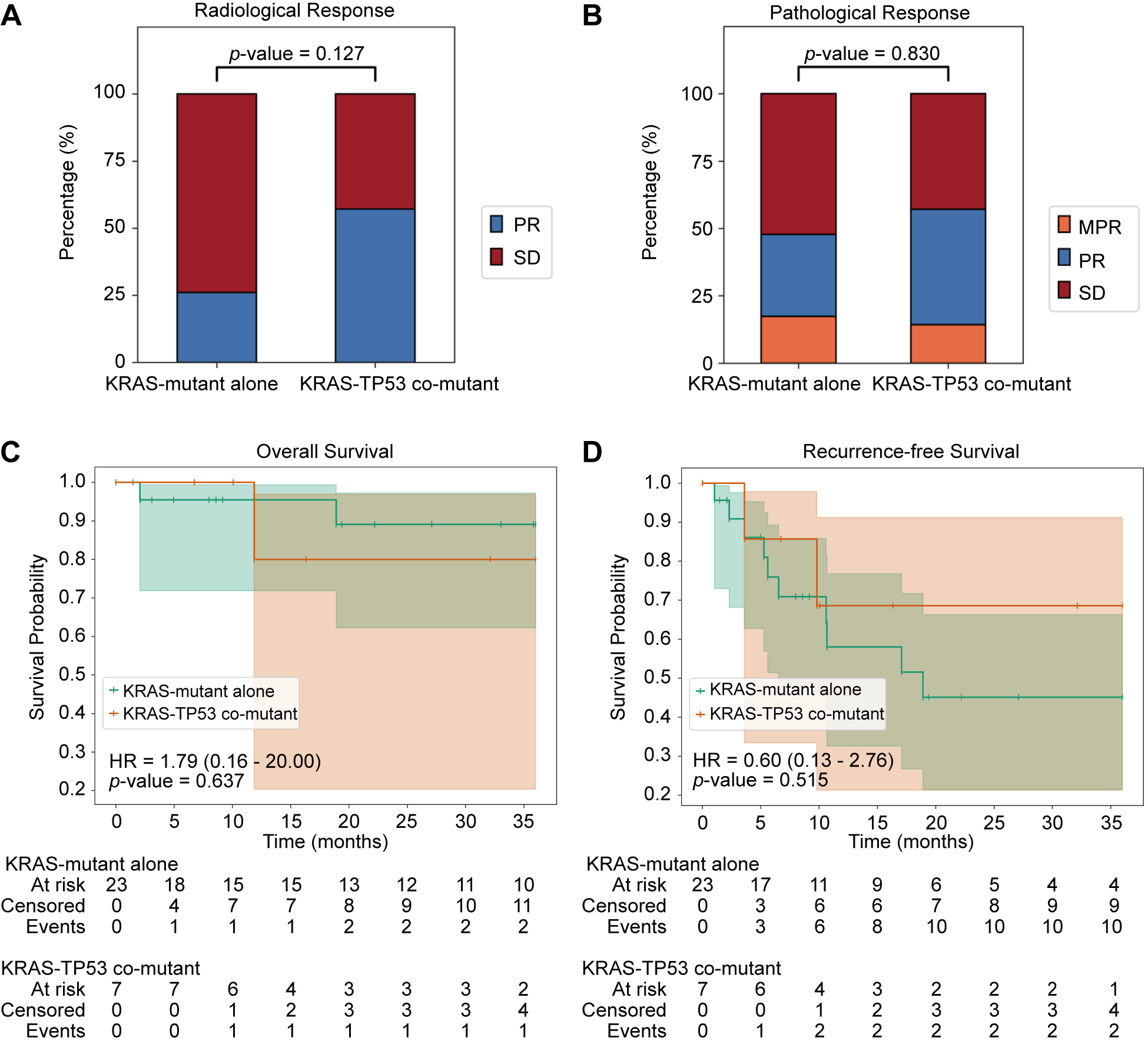

Supplement: Supplementary file 3 — Supporting Information [file CTM2-16-e70670-s010.tif]

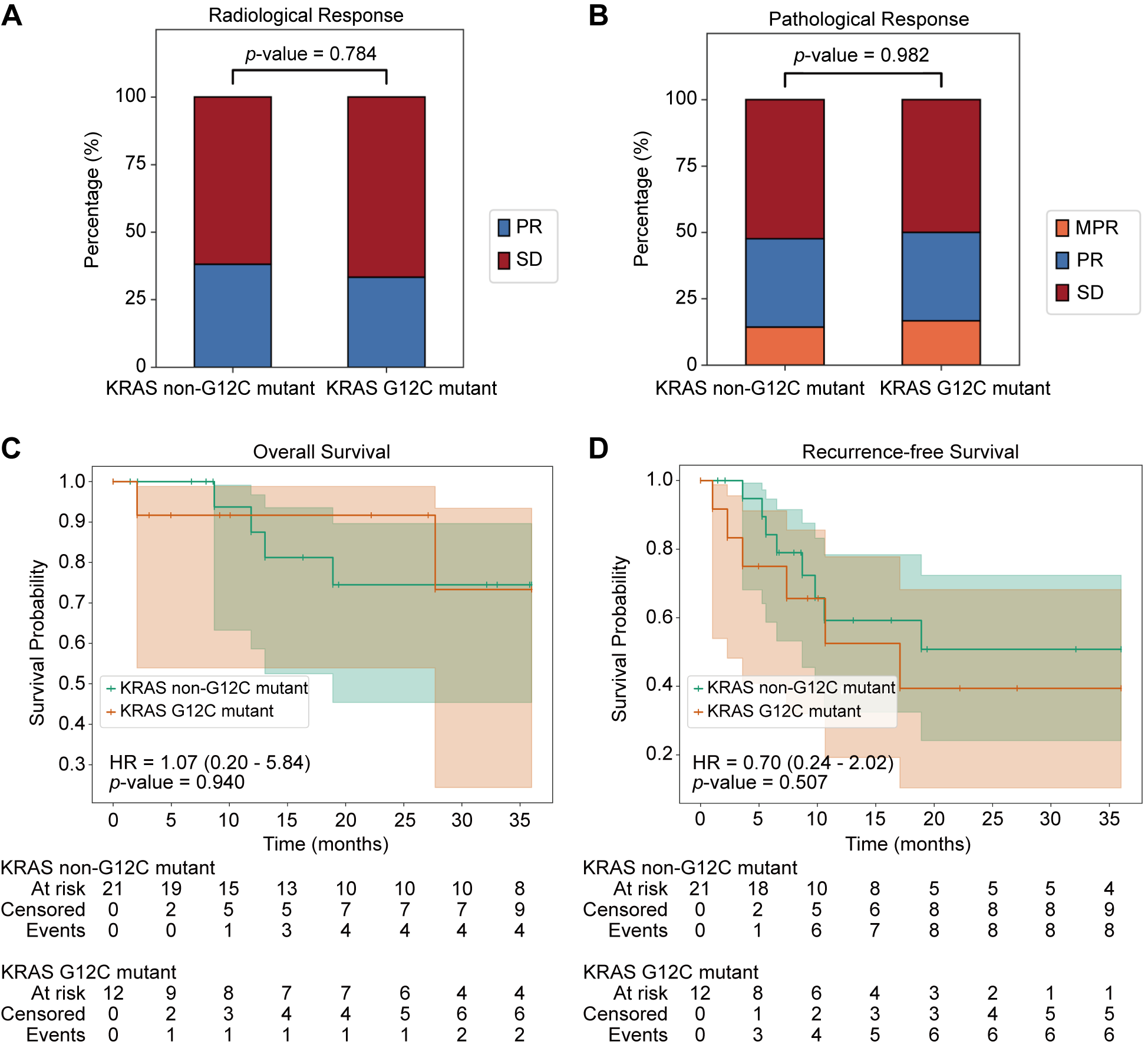

Supplement: Supplementary file 4 — Supporting Information [file CTM2-16-e70670-s014.tif]

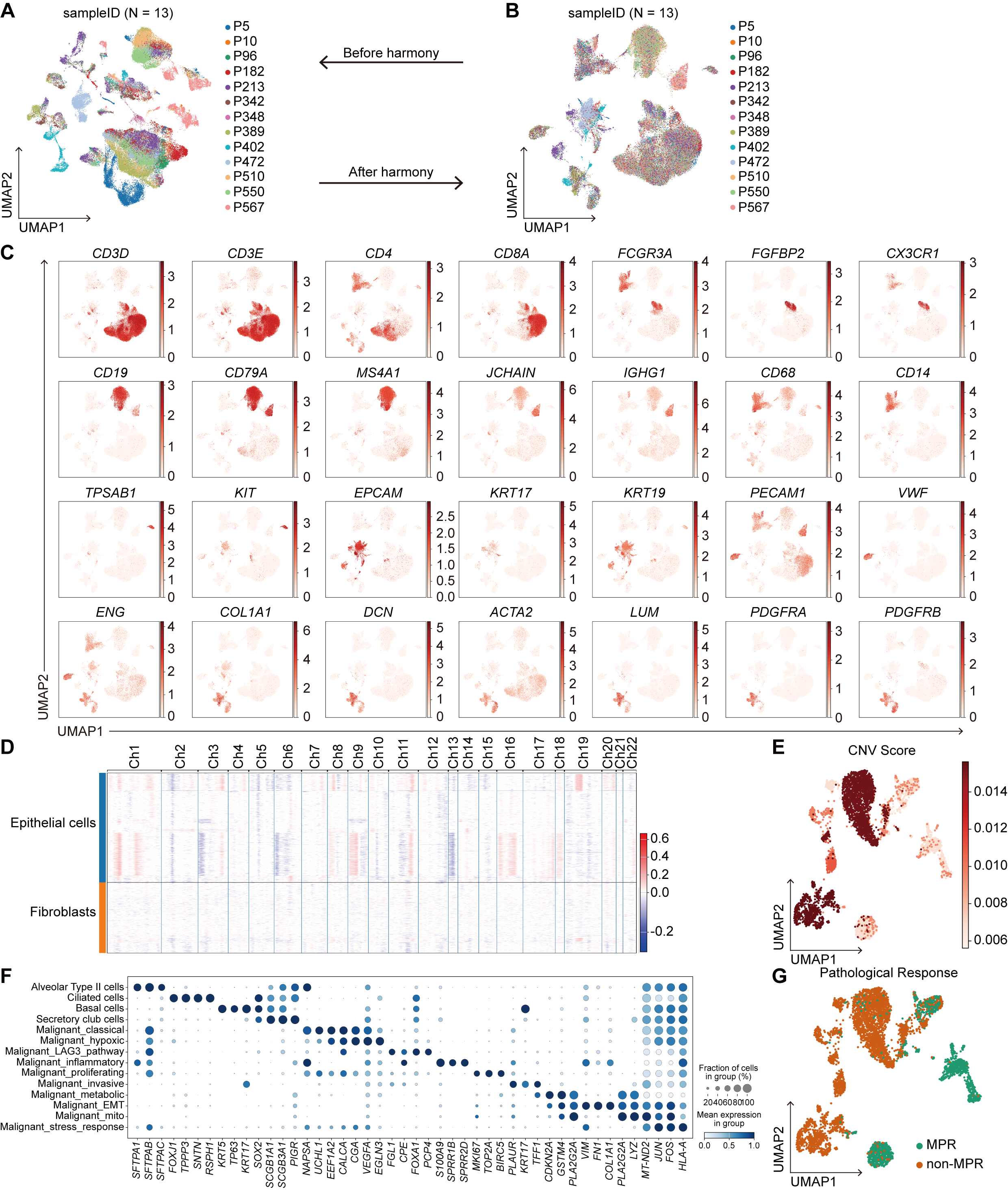

Supplement: Supplementary file 5 — Supporting Information [file CTM2-16-e70670-s009.tif]

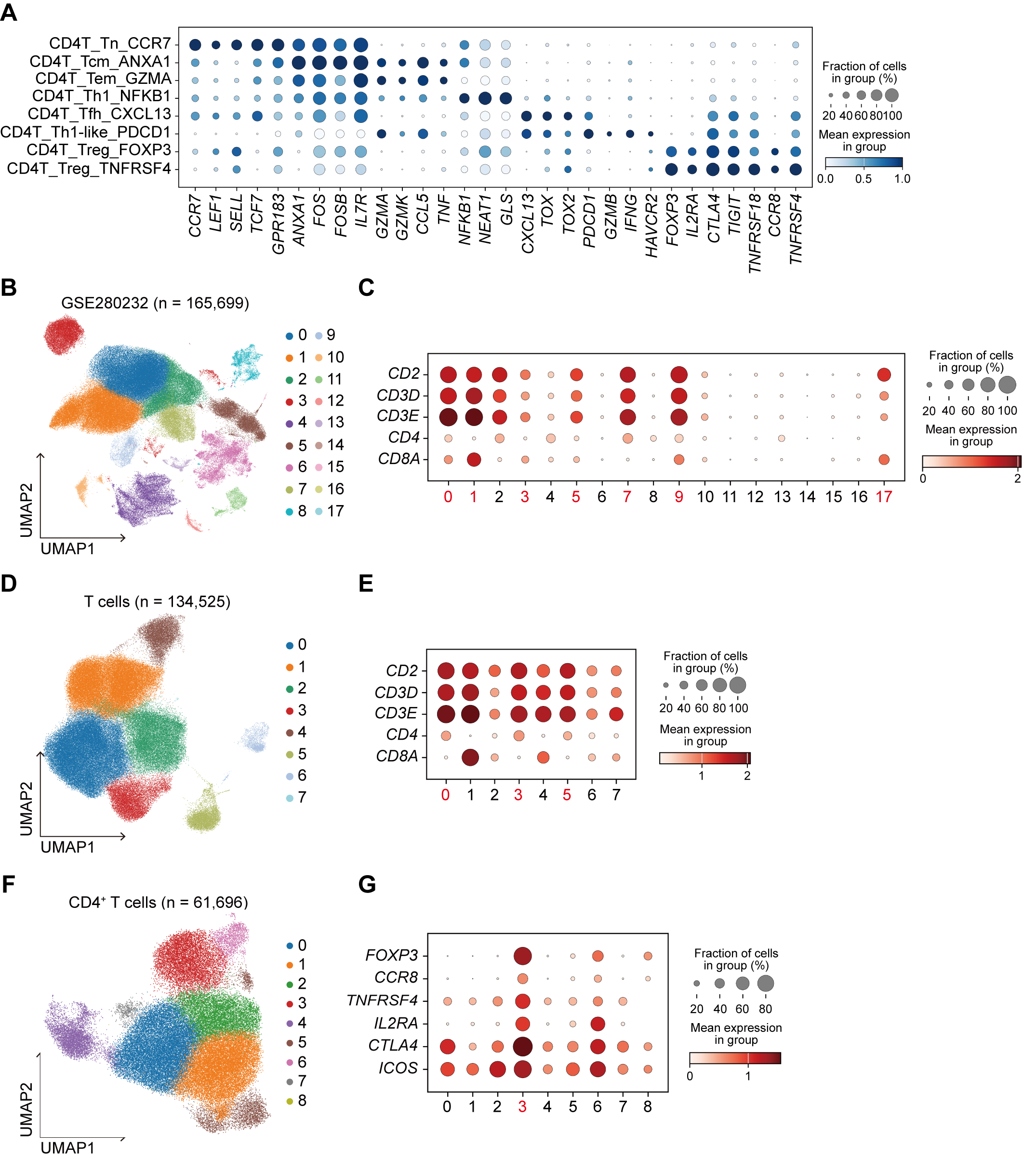

Supplement: Supplementary file 6 — Supporting Information [file CTM2-16-e70670-s008.tif]

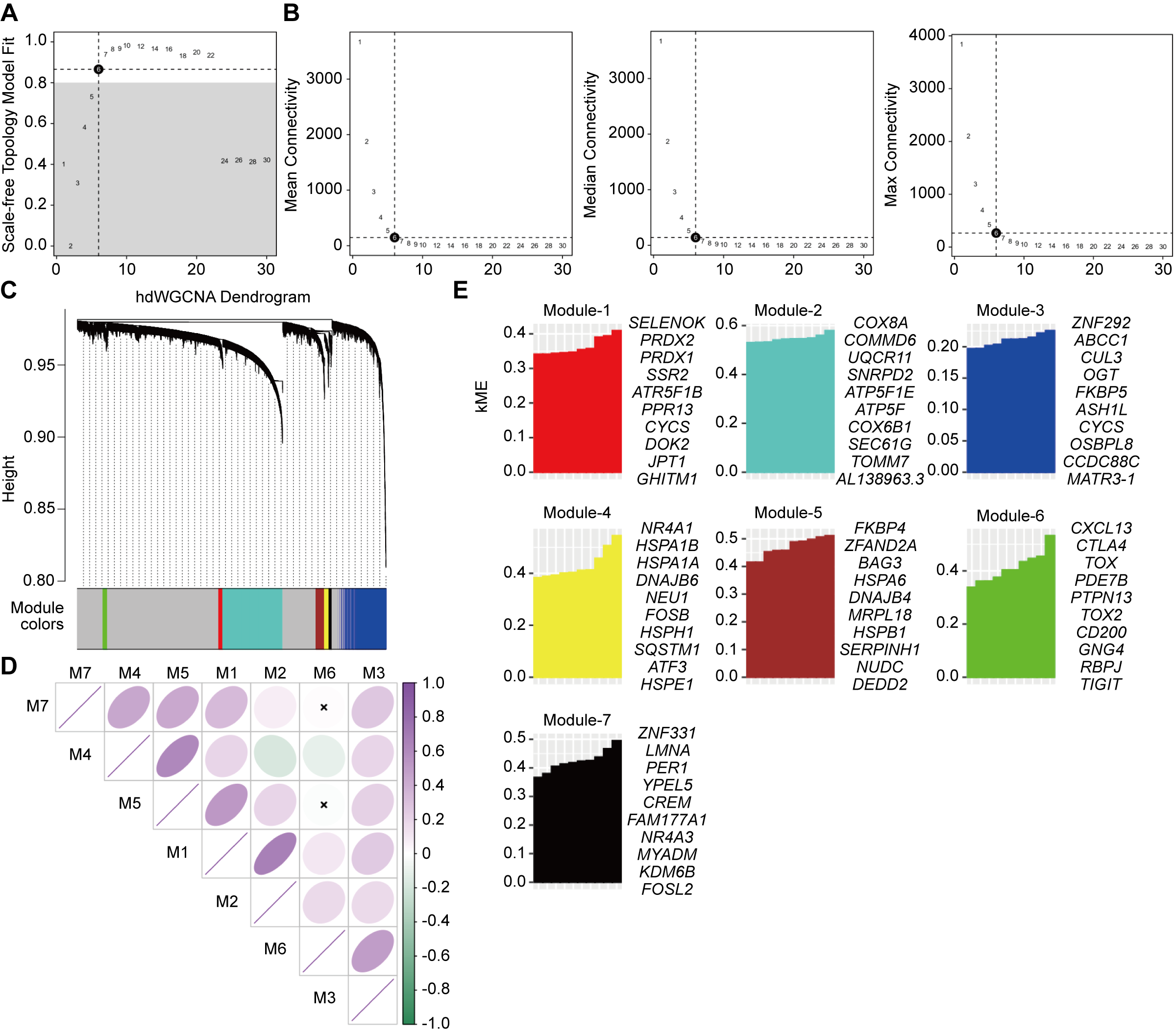

Supplement: Supplementary file 7 — Supporting Information [file CTM2-16-e70670-s015.tif]

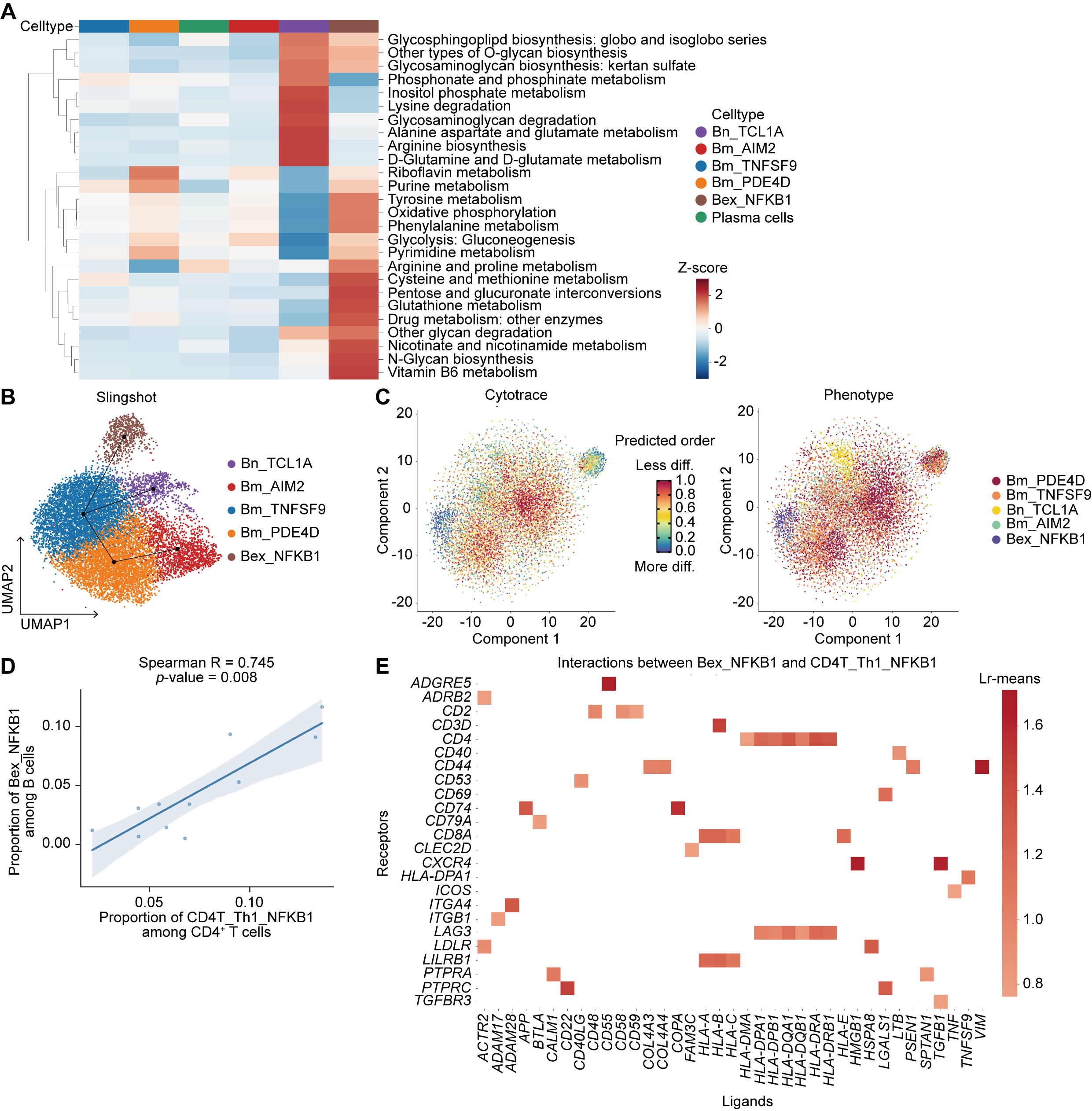

Supplement: Supplementary file 8 — Supporting Information [file CTM2-16-e70670-s013.tif]
