# Supplementary material for: The landscape of responses to neoadjuvant immunotherapy in resectable Kirsten rat sarcoma viral oncogene homolog‐mutant lung adenocarcinoma: Clinical heterogeneity and correlative immunologic analysis
Source: Clin Transl Med. 2026 Apr 20;16(4):e70670. doi: 10.1002/ctm2.70670 (PMC13096693; doi:10.1002/ctm2.70670)
Supplement: Supplementary file 9 — Supporting Information [file CTM2-16-e70670-s003.docx]

**Table S1.** Clinicopathological characteristics between patients with KRAS-mutant and KRAS-wildtype

| Characteristics | Before PSM | | |  |  | After PSM |  |
| --- | --- | --- | --- | --- | --- | --- | --- |
|  | KRAS-wildtype (n=106) | KRAS-mutant (n=37) | P value |  | KRAS-wildtype (n=99) | KRAS-mutant (n=33) | P value |
| Age, n (%) |  |  | 0.202 |  |  |  | 0.419 |
| ≤60 | 53 (50%) | 14 (37.8%) |  |  | 47 (47.5%) | 13 (39.4%) |  |
| >60 | 53 (50%) | 23 (62.2%) |  |  | 52 (52.5%) | 20 (60.6%) |  |
| Gender, n (%) |  |  | 0.221 |  |  |  | 0.426 |
| Male | 75 (70.8%) | 30 (81.1%) |  |  | 71 (71.7%) | 26 (78.8%) |  |
| Female | 31 (29.2%) | 7 (18.9%) |  |  | 28 (28.3%) | 7 (21.2%) |  |
| Surgical approach, n (%) |  |  | 0.024 |  |  |  | 0.103 |
| Thoracoscopic surgery | 76 (71.7%) | 19 (51.4%) |  |  | 72 (72.7%) | 19 (57.6%) |  |
| Open thoracotomy | 30 (28.3%) | 18 (48.6%) |  |  | 27 (27.3%) | 14 (42.4%) |  |
| BMI, kg/m^2^, n (%) |  |  | 0.092 |  |  |  | 0.131 |
| ≤24 | 46 (43.4%) | 22 (59.5%) |  |  | 42 (42.4%) | 19 (57.6%) |  |
| >24 | 60 (56.6%) | 15 (40.5%) |  |  | 57 (57.6%) | 14 (42.4%) |  |
| aCCI score, n (%) |  |  | 0.686 |  |  |  | 0.847 |
| 0 | 17 (16%) | 3 (8.1%) |  |  | 15 (15.2%) | 3 (9.1%) |  |
| 1 | 24 (22.6%) | 9 (24.3%) |  |  | 22 (22.2%) | 8 (24.2%) |  |
| 2 | 40 (37.7%) | 16 (43.2%) |  |  | 38 (38.4%) | 13 (39.4%) |  |
| ≥3 | 25 (23.6%) | 9 (24.3%) |  |  | 24 (24.2%) | 9 (27.3%) |  |
| Smoking history, n (%) |  |  | 0.080 |  |  |  | 0.226 |
| No | 52 (49.1%) | 12 (32.4%) |  |  | 48 (48.5%) | 12 (36.4%) |  |
| Yes | 54 (50.9%) | 25 (67.6%) |  |  | 51 (51.5%) | 21 (63.6%) |  |
| Family cancer history, n (%) |  |  | 0.073 |  |  |  | 0.152 |
| No | 77 (72.6%) | 21 (56.8%) |  |  | 73 (73.7%) | 20 (60.6%) |  |
| Yes | 29 (27.4%) | 16 (43.2%) |  |  | 26 (26.3%) | 13 (39.4%) |  |
| Tumor location, n (%) |  |  | 0.421 |  |  |  | 0.613 |
| Right upper lobe | 31 (29.2%) | 10 (27%) |  |  | 31 (31.3%) | 8 (24.2%) |  |
| Right middle lobe | 5 (4.7%) | 0 (0%) |  |  | 3 (3%) | 0 (0%) |  |
| Right lower lobe | 23 (21.7%) | 9 (24.3%) |  |  | 22 (22.2%) | 9 (27.3%) |  |
| Right middle-lower lobe | 5 (4.7%) | 0 (0%) |  |  | 3 (3%) | 0 (0%) |  |
| Left upper lobe | 23 (21.7%) | 12 (32.4%) |  |  | 21 (21.2%) | 10 (30.3%) |  |
| Left lower lobe | 19 (17.9%) | 6 (16.2%) |  |  | 19 (19.2%) | 6 (18.2%) |  |
| Type of resection, n (%) |  |  | 0.588 |  |  |  | 0.774 |
| Lobectomy | 94 (88.7%) | 31 (83.8%) |  |  | 88 (88.9%) | 28 (84.8%) |  |
| Sublobectomy | 2 (1.9%) | 0 (0%) |  |  | 1 (1%) | 0 (0%) |  |
| Sleeve lobectomy | 3 (2.8%) | 2 (5.4%) |  |  | 3 (3%) | 2 (6.1%) |  |
| Pneumonectomy | 7 (6.6%) | 4 (10.8%) |  |  | 7 (7.1%) | 3 (9.1%) |  |
| Clinical stage, n (%) |  |  | 0.580 |  |  |  | 0.622 |
| IIA | 4 (3.8%) | 0 (0%) |  |  | 2 (2%) | 0 (0%) |  |
| IIB | 25 (23.6%) | 11 (29.7%) |  |  | 22 (22.2%) | 10 (30.3%) |  |
| IIIA | 55 (51.9%) | 19 (51.4%) |  |  | 54 (54.5%) | 16 (48.5%) |  |
| IIIB | 19 (17.9%) | 7 (18.9%) |  |  | 18 (18.2%) | 7 (21.2%) |  |
| IIIC | 3 (2.8%) | 0 (0%) |  |  | 3 (3%) | 0 (0%) |  |
| Pathological stage, n (%) |  |  | 0.257 |  |  |  | 0.440 |
| O | 14 (13.2%) | 1 (2.7%) |  |  | 12 (12.1%) | 1 (3%) |  |
| IA | 31 (29.2%) | 9 (24.3%) |  |  | 29 (29.3%) | 9 (27.3%) |  |
| IB | 11 (10.4%) | 4 (10.8%) |  |  | 11 (11.1%) | 3 (9.1%) |  |
| IIA | 5 (4.7%) | 0 (0%) |  |  | 3 (3%) | 0 (0%) |  |
| IIB | 10 (9.4%) | 7 (18.9%) |  |  | 10 (10.1%) | 7 (21.2%) |  |
| IIIA | 26 (24.5%) | 12 (32.4%) |  |  | 25 (25.3%) | 9 (27.3%) |  |
| IIIB | 9 (8.5%) | 4 (10.8%) |  |  | 9 (9.1%) | 4 (12.1%) |  |
| PD-L1 expression level |  |  | 0.060 |  |  |  | 0.154 |
| <1% | 18 (17%) | 13 (35.1%) |  |  | 18 (18.2%) | 12 (36.4%) |  |
| 1%-49% | 23 (21.7%) | 6 (16.2%) |  |  | 20 (20.2%) | 6 (18.2%) |  |
| ≥50% | 11 (10.4%) | 6 (16.2%) |  |  | 11 (11.1%) | 4 (12.1%) |  |
| Unknown | 54 (50.9%) | 12 (32.4%) |  |  | 50 (50.5%) | 11 (33.3%) |  |
| Visceral pleural invasion, n (%) |  |  | 0.213 |  |  |  | 0.123 |
| No | 85 (80.2%) | 26 (70.3%) |  |  | 79 (79.8%) | 22 (66.7%) |  |
| Yes | 21 (19.8%) | 11 (29.7%) |  |  | 20 (20.2%) | 11 (33.3%) |  |
| Spread through air space, n (%) |  |  | 0.237 |  |  |  | 0.348 |
| No | 82 (77.4%) | 25 (67.6%) |  |  | 77 (77.8%) | 23 (69.7%) |  |
| Yes | 24 (22.6%) | 12 (32.4%) |  |  | 22 (22.2%) | 10 (30.3%) |  |
| Vascular invasion, n (%) |  |  | 0.937 |  |  |  | 1.000 |
| No | 95 (89.6%) | 34 (91.9%) |  |  | 89 (89.9%) | 30 (90.9%) |  |
| Yes | 11 (10.4%) | 3 (8.1%) |  |  | 10 (10.1%) | 3 (9.1%) |  |
| Nerve invasion, n (%) |  |  | 0.937 |  |  |  | 1.000 |
| No | 95 (89.6%) | 34 (91.9%) |  |  | 89 (89.9%) | 30 (90.9%) |  |
| Yes | 11 (10.4%) | 3 (8.1%) |  |  | 10 (10.1%) | 3 (9.1%) |  |
| Tumor thrombosis, n (%) |  |  | 0.686 |  |  |  | 0.895 |
| No | 89 (84%) | 30 (81.1%) |  |  | 82 (82.8%) | 27 (81.8%) |  |
| Yes | 17 (16%) | 7 (18.9%) |  |  | 17 (17.2%) | 6 (18.2%) |  |
| Adjuvant therapy, n (%) |  |  | 0.928 |  |  |  | 0.727 |
| No | 9 (8.5%) | 4 (10.8%) |  |  | 8 (8.1%) | 4 (12.1%) |  |
| Yes | 97 (91.5%) | 33 (89.2%) |  |  | 91 (91.9%) | 29 (87.9%) |  |

Footnote: aCCI, age-adjusted Charlson Comorbidity Index; BMI, body mass index; KRAS, Kirsten Rat Sarcoma Viral Oncogene Homolog; PD-L1, Programmed Cell Death Ligand 1; PSM, propensity score matching.

**Table S2.** Characteristics of the perioperative outcomes and postoperative pathological response between patients with KRAS-mutant and KRAS-wildtype after PSM

| Characteristics | KRAS-mutant (n=33) | KRAS-wildtype (n=99) | P value |
| --- | --- | --- | --- |
| Operation time, min, median (IQR) | 133 (107, 152) | 143 (114.5, 174) | 0.131 |
| Intraoperatve blood loss, mL, median (IQR) | 10 (10, 20) | 10 (10, 20) | 0.165 |
| ICU stay after surgery, n (%) |  |  | 1.000 |
| No | 32 (97%) | 98 (99%) |  |
| Yes | 1 (3%) | 1 (1%) |  |
| Postoperative hospital stay, day, median (IQR) | 5 (4, 6) | 4 (4, 7) | 0.723 |
| Postoperative complications, n (%) |  |  | 1.000 |
| No | 32 (97%) | 95 (96%) |  |
| Yes | 1 (3%) | 4 (4%) |  |
| KRAS mutation subtype |  |  | - |
| G12C | 12 (36.4%) | - |  |
| G12D | 8 (24.2%) | - |  |
| G12V | 4 (12.1%) | - |  |
| Other | 3 (9.1%) | - |  |
| G12R | 1 (3%) | - |  |
| Q61H | 3 (9.1%) | - |  |
| G13D | 1 (3%) | - |  |
| G12A | 1 (3%) |  |  |
| The co-mutation, n (%) |  |  | - |
| No | 23 (69.7%) | - |  |
| TP53 | 7 (21.2%) | - |  |
| STK11 | 3 (9.1%) | - |  |
| KEAP1 | 0 (0.0%) | - |  |
| Pathological response, n (%) |  |  | 0.032 |
| SD | 17 (51.5%) | 25 (25.3%) |  |
| PR | 11 (33.3%) | 43 (43.4%) |  |
| MPR | 5 (15.2%) | 27 (27.3%) |  |
| pCR | 0 (0%) | 4 (4%) |  |
| Radiological response, n (%) |  |  | 0.070 |
| SD | 21 (63.6%) | 45 (45.5%) |  |
| PR | 12 (36.4%) | 54 (54.5%) |  |
| Number of lymph nodes dissected, median (Mean±SD) | 24 (17, 31) | 20 (14, 27.5) | 0.243 |
| Number of lymph node metastasis, median (IQR) | 0 (0, 2) | 0 (0, 1) | 0.701 |
| Number of N2 lymph nodes dissected, median (IQR) | 11 (8, 16) | 10 (7, 16) | 0.586 |
| Number of N2 lymph node metastasis, median (IQR) | 0 (0, 1) | 0 (0, 1) | 0.803 |
| T-stage down-staging , n (%) |  |  | 0.916 |
| No | 12 (36.4%) | 35 (35.4%) |  |
| Yes | 21 (63.6%) | 64 (64.6%) |  |
| N-stage down-staging, n (%) |  |  | 0.070 |
| No | 22 (66.7%) | 48 (48.5%) |  |
| Yes | 11 (33.3%) | 51 (51.5%) |  |
| CEA-Post NIT, median (IQR) | 4.33 (2.82, 6.84) | 3.47 (2.16, 5.57) | 0.326 |

Footnote: ICU, intensive care unit; MPR, major pathological response; pCR, completed pathological response; SD, stable disease; PR, partial response; CR, completed response; CEA-Post NIT, carcinoembryonic antigen-post neoadjuvant immunotherapy; PSM, propensity score matching.

**Table S3.** Overall and recurrence-free survival Cox proportional hazards model in the overall cohort

|  |  | 3-years OS | | | | |  | 3-years RFS | | | | |
| --- | --- | --- | --- | --- | --- | --- | --- | --- | --- | --- | --- | --- |
| Characteristics | Total  (N=143) | Univariate Analysis | |  | Multivariate analysis | |  | Univariate Analysis | |  | Multivariate analysis | |
|  |  | Hazard ratio  (95% CI) | P value |  | Hazard ratio  (95% CI) | P value |  | Hazard ratio  (95% CI) | P value |  | Hazard ratio  (95% CI) | P value |
| KRAS status |  |  |  |  |  |  |  |  |  |  |  |  |
| KRAS-wildtype | 106 | Reference |  |  | Reference |  |  | Reference |  |  | Reference |  |
| KRAS-mutant | 37 | 5.01 (1.59 - 15.81) | **0.006** |  | 14.21 (2.88 - 70.03) | **0.001** |  | 2.33 (1.22 - 4.44) | **0.010** |  | 2.55 (1.24 - 5.25) | **0.011** |
| Co-mutant status |  |  |  |  |  |  |  |  |  |  |  |  |
| No | 106 | Reference |  |  | Reference |  |  | Reference |  |  | - | - |
| KRAS mutant alone | 27 | 2.79 (0.67 - 11.67) | 0.161 |  | 2.76 (0.66 - 11.65) | 0.166 |  | 2.37 (1.16 - 4.83) | **0.018** |  | - | - |
| KRAS-STK11 co-mutant | 3 | 39.19 (8.86 - 173.31) | **< 0.001** |  | 43.76 (7.03 - 272.46) | **< 0.001** |  | 4.84 (1.12 - 21.01) | **0.035** |  | - | - |
| KRAS-TP53  co-mutant | 7 | 4.22 (0.49 - 36.22) | 0.190 |  | 1.00 (0.12 - 8.28) | 1.000 |  | 1.47 (0.35 - 6.22) | 0.600 |  | - | - |
| Age, n (%) |  |  |  |  |  |  |  |  |  |  |  |  |
| ≤60 | 67 | Reference |  |  | - | - |  | Reference |  |  | - | - |
| >60 | 76 | 1.91 (0.58 - 6.35) | 0.291 |  | - | - |  | 1.18 (0.64 - 2.21) | 0.596 |  | - | - |
| Gender, n (%) |  |  |  |  |  |  |  |  |  |  |  |  |
| Male | 105 | Reference |  |  | - | - |  | Reference |  |  | - | - |
| Female | 38 | 0.19 (0.02 - 1.45) | 0.108 |  | - | - |  | 0.96 (0.48 - 1.92) | 0.901 |  | - | - |
| Smoking history, n (%) |  |  |  |  |  |  |  |  |  |  |  |  |
| No | 64 | Reference |  |  | - | - |  | Reference |  |  | - | - |
| Yes | 79 | 3.32 (0.89 - 12.31) | 0.073 |  | - | - |  | 0.77 (0.41 - 1.44) | 0.412 |  | - | - |
| Family cancer history, n (%) |  |  |  |  |  |  |  |  |  |  |  |  |
| No | 98 | Reference |  |  | - | - |  | Reference |  |  | - | - |
| Yes | 45 | 1.55 (0.49 - 4.89) | 0.453 |  | - | - |  | 1.54 (0.82 - 2.91) | 0.178 |  | - | - |
| BMI, n (%) |  |  |  |  |  |  |  |  |  |  |  |  |
| ≤24 | 68 | Reference |  |  | Reference |  |  | Reference |  |  | - | - |
| >24 | 75 | 0.19 (0.04 - 0.86) | **0.031** |  | 0.20 (0.04 - 1.06) | 0.059 |  | 0.74 (0.40 - 1.38) | 0.347 |  | - | - |
| Pathological stage |  | Reference |  |  |  |  |  |  |  |  |  |  |
| O | 15 | - | - |  | - | - |  | Reference |  |  | Reference |  |
| IA | 40 | - | - |  | - | - |  | 2.39 (0.29 - 19.86) | 0.420 |  | 1.88 (0.22 - 16.28) | 0.566 |
| IB | 15 | - | - |  | - | - |  | 2.29 (0.21 - 25.32) | 0.498 |  | 0.77 (0.052 - 11.20) | 0.846 |
| IIA | 5 | - | - |  | - | - |  | 10.47 (0.94 - 116.19) | 0.056 |  | 9.85 (0.82 - 118.33) | 0.071 |
| IIB | 17 | - | - |  | - | - |  | 10.54 (1.32 - 84.39) | **0.026** |  | 4.76 (0.52 - 43.81) | 0.169 |
| IIIA | 38 | - | - |  | - | - |  | 7.85 (1.04 - 59.23) | **0.046** |  | 4.16 (0.50 - 34.75) | 0.188 |
| IIIB | 13 | - | - |  | - | - |  | 7.78 (0.91 - 66.64) | 0.061 |  | 3.36 (0.34 - 33.72) | 0.303 |
| Surgical approach, n (%) |  |  |  |  |  |  |  |  |  |  |  |  |
| Thoracoscopic surgery | 95 | Reference |  |  | - | - |  | Reference |  |  | - | - |
| Open thoracotomy | 48 | 3.14 (1.00 - 9.91) | 0.051 |  | - | - |  | 1.52 (0.80 - 2.86) | 0.198 |  | - | - |
| Type of resection, n (%) |  |  |  |  |  |  |  |  |  |  |  |  |
| Lobectomy | 125 | Reference |  |  | Reference |  |  | Reference |  |  | Reference |  |
| Sublobectomy | 2 | - | - |  | - | - |  | - | - |  | - | - |
| Sleeve Lobectomy | 5 | 10.43 (1.17 - 93.00) | **0.036** |  | 6.43 (0.61 - 67.87) | 0.122 |  | 1.22 (0.17 - 8.98) | 0.845 |  | 3.05 (0.37 - 25.36) | 0.303 |
| Pneumonectomy | 11 | 6.83 (2.00 - 23.38) | **0.002** |  | 28.47(4.43- 183.20) | **< 0.001** |  | 3.11 (1.37 - 7.07) | **0.007** |  | 4.46 (1.66 - 11.98) | **0.003** |
| Visceral Pleural Invasion, n (%) | 143 |  |  |  |  |  |  |  |  |  |  |  |
| No | 111 | Reference |  |  | - | - |  | Reference |  |  | - | - |
| Yes | 32 | 2.31 (0.73 - 7.27) | 0.154 |  | - | - |  | 1.94 (1.00 - 3.76) | 0.051 |  | - | - |
| Spread through air space, n (%) |  |  |  |  |  |  |  |  |  |  |  |  |
| No | 107 | Reference |  |  | - | - |  | Reference |  |  | - | - |
| Yes | 36 | 1.55 (0.47 - 5.16) | 0.473 |  | - | - |  | 0.87 (0.41 - 1.82) | 0.707 |  | - | - |
| Vascular invasion, n (%) |  |  |  |  |  |  |  |  |  |  |  |  |
| No | 129 | Reference |  |  | - | - |  | Reference |  |  | - | - |
| Yes | 14 | 1.74 (0.38 - 7.93) | 0.476 |  | - | - |  | 1.28 (0.50 - 3.28) | 0.601 |  | - | - |
| Nerve invasion, n (%) |  |  |  |  |  |  |  |  |  |  |  |  |
| No | 129 | Reference |  |  | - | - |  | Reference |  |  | - | - |
| Yes | 14 | 1.86 (0.41 - 8.48) | 0.426 |  | - | - |  | 2.17 (0.96 - 4.90) | 0.064 |  | - | - |
| Tumor thrombosis, n (%) |  |  |  |  |  |  |  |  |  |  |  |  |
| No | 119 | Reference |  |  | - | - |  | Reference |  |  | - | - |
| Yes | 24 | 2.20 (0.66 - 7.31) | 0.198 |  | - | - |  | 1.68 (0.82 - 3.45) | 0.153 |  | - | **-** |
| Operation time, min, median (IQR) | 143 | 1.00 (0.99 - 1.01) | 0.952 |  | - | - |  | 1.00 (0.99 - 1.01) | 0.548 |  |  |  |
| Intraoperatve blood loss, median (IQR) | 143 | 1.00 (0.99 - 1.02) | 0.775 |  | - | - |  | 1.00 (0.98 - 1.01) | 0.503 |  | - | - |
| Postoperative hospital stay, median (IQR) | 143 | 1.11 (0.85 - 1.45) | 0.427 |  | - | - |  | 1.08 (0.93 - 1.24) | 0.312 |  | - | - |
| Number of lymph nodes dissected, median (Mean±SD) | 143 | 1.02 (0.98 - 1.07) | 0.369 |  | - | - |  | 1.00 (0.97 - 1.03) | 0.769 |  | - | - |
| Number of lymph node metastasis, median (IQR) | 143 | 1.07 (0.98 - 1.17) | 0.127 |  | - | - |  | 1.07 (1.02 - 1.13) | **0.009** |  | 0.96 (0.82 - 1.14) | 0.653 |
| Number of N2 lymph nodes dissected, median (IQR) | 143 | 0.97 (0.88 - 1.06) | 0.467 |  | - | - |  | 0.97 (0.93 - 1.02) | 0.252 |  | - | - |
| Number of N2 lymph node metastasis, median (IQR) | 143 | 1.08 (0.87 - 1.34) | 0.473 |  | - | - |  | 1.18 (1.04 - 1.33) | **0.008** |  | 1.31 (0.94 - 1.82) | 0.109 |
| CEA-Post NIT, median (IQR) | 102 | 1.03 (1.01 - 1.05) | **0.011** |  | 1.01 (0.98 - 1.03) | 0.611 |  | 1.01 (0.99 - 1.04) | 0.271 |  | - | - |
| Adjuvant therapy, n (%) |  |  |  |  |  |  |  |  |  |  |  |  |
| No | 13 | Reference |  |  | - | - |  | Reference |  |  | - | - |
| immunotherapy | 32 | 0.89 (0.09 - 8.58) | 0.920 |  | - | - |  | 0.42 (0.13 - 1.34) | 0.143 |  | - | - |
| Chemotherapy | 17 | 0.51 (0.03 - 8.16) | 0.633 |  | - | - |  | 0.60 (0.17 - 2.08) | 0.422 |  | - | - |
| Immunochemotherapy | 71 | 1.11 (0.13 - 9.54) | 0.923 |  | - | - |  | 0.68 (0.25 - 1.86) | 0.456 |  | - | - |
| Other | 10 | 1.94 (0.18 - 21.49) | 0.588 |  | - | - |  | 1.93 (0.59 - 6.32) | 0.280 |  | - | - |

Footnote: BMI, body mass index; CEA-Post NIT, carcinoembryonic antigen-post neoadjuvant immunotherapy; KRAS, Kirsten rats arcomaviral oncogene homolog; NIT, neoadjuvant immunotherapy.

**Table S4.** Recurrence pattern between the KRAS-wildtype group and the KRAS-mutant group.

| Location | Recurrence | | P value |
| --- | --- | --- | --- |
|  | KRAS-mutant  (14/37) | KRAS-wildtype (25/106) |  |
| **Loco-regional recurrence** |  |  | 0.960 |
| Mediastinal LN, n (%) | 0 (0.0%) | 4 (3.8%) | 0.536 |
| Bronchial stump, n (%) | 0 (0.0%) | 1 (0.9%) | 1.000 |
| Ipsilateral Lung, n (%) | 1 (2.7%) | 0 (0.0%) | 0.259 |
| **Distant recurrence** |  |  | 0.043 |
| Supraclavicular LN, n (%) | 0 (0.0%) | 2 (1.9%) | 0.977 |
| Pleural, n (%) | 1 (2.7%) | 3 (2.8%) | 1.000 |
| Liver, n (%) | 0 (0.0%) | 2 (1.9%) | 0.977 |
| Brain, n (%) | 4 (10.8%) | 4 (3.8%) | 0.235 |
| Contralateral Lung, n (%) | 3 (8.1%) | 1 (0.9%) | 0.090 |
| Bone | 2 (5.4%) | 0 (0.0%) | 0.110 |
| Multiple organs, n (%) | 3 (8.1%) | 6 (5.7%) | 0.893 |
| Other, n (%) | 0 (4.0%) | 2 (1.9%) | 0.977 |

Abbreviation: LN, lymph node; KRAS, Kirsten rats arcomaviral oncogene homolog.
